# Dietary intake of total, animal, and plant proteins and risk of all cause, cardiovascular, and cancer mortality: systematic review and dose-response meta-analysis of prospective cohort studies

**DOI:** 10.1136/bmj.m2412

**Published:** 2020-07-22

**Authors:** Sina Naghshi, Omid Sadeghi, Walter C Willett, Ahmad Esmaillzadeh

**Affiliations:** 1 Students’ Scientific Research Center, Tehran University of Medical Sciences, Tehran, Iran; 2 Department of Clinical Nutrition, School of Nutritional Sciences and Dietetics, Tehran University of Medical Sciences, Tehran, Iran; 3 Department of Community Nutrition, School of Nutritional Sciences and Dietetics, Tehran University of Medical Sciences, Tehran, Iran; 4 Departments of Nutrition and Epidemiology, Harvard TH Chan School of Public Health, Boston, MA, USA; 5 Channing Division of Network Medicine, Brigham and Women’s Hospital and Harvard Medical School, Boston, MA, USA; 6 Department of Community Nutrition, School of Nutritional Sciences and Dietetics, Tehran University of Medical Sciences, PO Box 14155-6117, Tehran, Iran; 7 Obesity and Eating Habits Research Centre, Endocrinology and Metabolism Molecular-Cellular Sciences Institute, Tehran University of Medical Sciences, Tehran, Iran; 8 Department of Community Nutrition, School of Nutrition and Food Science, Isfahan University of Medical Sciences, Isfahan, Iran

## Abstract

**Objective:**

To examine and quantify the potential dose-response relation between intake of total, animal, and plant protein and the risk of mortality from all causes, cardiovascular disease, and cancer.

**Design:**

Systematic review and meta-analysis of prospective cohort studies.

**Data sources:**

PubMed, Scopus, and ISI Web of Science until December 2019, and references of retrieved relevant articles.

**Study selection:**

Prospective cohort studies that reported the risk estimates for all cause, cardiovascular, and cancer mortality in adults aged 18 or older.

**Data synthesis:**

Random effects models were used to calculate pooled effect sizes and 95% confidence intervals for the highest versus lowest categories of protein intake and to incorporate variation between studies. Linear and non-linear dose-response analyses were done to evaluate the dose-response relations between protein intake and mortality.

**Results:**

32 prospective cohort studies were included in the systematic review and 31 in the meta-analysis. During the follow-up period of 3.5 to 32 years, 113 039 deaths (16 429‬ from cardiovascular disease and 22 303‬ from cancer) occurred among 715 128 participants. Intake of total protein was associated with a lower risk of all cause mortality (pooled effect size 0.94, 95% confidence interval 0.89 to 0.99, I^2^=58.4%, P<0.001). Intake of plant protein was significantly associated with a lower risk of all cause mortality (pooled effect size 0.92, 95% confidence interval 0.87 to 0.97, I^2^=57.5%, P=0.003) and cardiovascular disease mortality (pooled hazard ratio 0.88, 95% confidence interval 0.80 to 0.96, I^2^=63.7%, P=0.001), but not with cancer mortality. Intake of total and animal protein was not significantly associated with risk of cardiovascular disease and cancer mortality. A dose-response analysis showed a significant inverse dose-response association between intake of plant protein and all cause mortality (P=0.05 for non-linearity). An additional 3% energy from plant proteins a day was associated with a 5% lower risk of death from all causes.

**Conclusions:**

Higher intake of total protein was associated with a lower risk of all cause mortality, and intake of plant protein was associated with a lower risk of all cause and cardiovascular disease mortality. Replacement of foods high in animal protein with plant protein sources could be associated with longevity.

## Introduction

Cardiovascular disease and cancer are two leading causes of death, contributing to 26.9 million deaths worldwide in 2016.^1^ Diet has an important role in these conditions. The optimal macronutrient composition of a diet for supporting longevity remains uncertain,^2^ ^3^ particularly for protein intake. A global transition towards higher protein diets has occurred in recent decades.^4^ In addition, adherence to a high protein diet has recently become popular because of its possible effects on weight loss, preservation of muscle mass, and increased strength.^5^ ^6^

High protein diets have also been linked to improvements in cardiometabolic biomarkers, including blood glucose and blood pressure levels. Increasing evidence suggests that diets rich in protein, particularly protein from plants, significantly****decrease serum concentrations of blood lipids, without any significant effect on concentrations of high density lipoprotein cholesterol and the risk of cardiovascular disease.^7^ These effects may be related to bioactive peptides and the amino acid composition of plant proteins, but other components in the same foods could also contribute. A significant positive association between animal protein intake and an increased incidence of cardiovascular disease and some cancers has also been reported,^8^ which could be attributed to the content of high sulfur amino acids in animal proteins.

Findings on the association between total protein intake and longevity are still controversial. Total protein intake was associated with a decreased risk of mortality in some investigations,^9^ ^10^ but others failed to find such evidence.^11^ ^12^ The same findings have also been reported for animal or plant proteins.^11^ ^13^ ^14^ Several studies found that consumption of animal proteins was associated with a higher risk of mortality,^15^ ^16^ ^17^ whereas others reported no significant association between intake of animal or plant proteins and risk of all cause and cause specific mortality.^11^ ^13^ ^18^ A recent meta-analysis showed that intake of soy protein was associated with a reduced risk of breast cancer mortality, but it was not associated with all cause and cardiovascular disease mortality.^19^ No information is available for the strength and shape of a dose-response relation between consumption of proteins and risk of mortality. We conducted a systematic review and dose-response meta-analysis of prospective cohort studies to summarise the association between intake of dietary protein and risk of mortality from all causes, cardiovascular disease, and cancer.

## Methods

Findings from this systematic review and meta-analysis were reported based on the preferred reporting items for systematic review and meta-analysis (PRISMA) guideline.^20^

### Search strategy

We conducted a systematic search of all articles published up to 31 December 2019 of online databases, including PubMed/Medline, ISI Web of Science, and Scopus, with no limitation on language or time of publication. Supplementary table 1 provides details of the search terms. To avoid missing any publication, we also checked the reference lists of extracted papers and recent reviews. Unpublished studies were not included because they could have been of lower methodological quality than published studies owing to the absence of peer review.^21^ Duplicate citations were removed.

### Inclusion and exclusion criteria

Published studies were included if they were observational prospective studies conducted on human adults, or studies that reported effect sizes including hazard ratios or relative risks or odds ratios with the corresponding 95% confidence intervals for the association between intake of total protein, animal protein, or plant protein as the exposure of interest and mortality from all causes, cardiovascular disease, total or specific cancers as the outcome of interest. All outcomes were classified based on the World Health Organization’s ICD-10 (international classification of diseases, 10th revision).^22^ If the same dataset had been published in more than one publication, we included the one with more complete findings or the greatest number of participants.

We excluded letters, comments, reviews, meta-analyses, and ecological studies. We also excluded studies performed on children or adolescents and on patients with chronic kidney disease or who were undergoing haemodialysis, end stage cancer, or critical illness. In addition, studies that considered urine urea nitrogen, as a surrogate index of protein intake, and those that considered individual dietary sources of protein as the exposure, rather than total protein, were excluded. If a study reported the effect sizes for risk of disease and mortality combined, we did not include it in the analysis. Moreover, studies with insufficient data were excluded, as were studies on protein intake from specific sources such as soy or legumes.

### Data extraction

Two researchers (SN and OS) conducted data extraction independently and resolved any disagreements in consultation with the principal investigator (AE). From each eligible article we extracted the name of the first author, publication year, study design, location of study, age range and health status at study entry, sex, cohort size, incidence of death, duration of follow-up, exposure, method used for assessment of exposure, comparison categories, and relevant effect sizes of comparison categories together with 95% confidence intervals and confounding variables adjusted for in the statistical analysis. When the data were reported for men and women separately, we considered each part as a distinct study. If an included study reported several risk estimates, we extracted the fully adjusted effect sizes. Numerical estimates were extracted from graphs using Plot Digitizer (http://plotdigitizer.sourceforge.net/).

### Risk of bias assessment

Risk of bias was assessed using the non-randomised studies of exposures (ROBINS-E) tool.^23^ This tool comprises seven domains—bias due to confounding, departure from intended exposures, and missing data, and bias in the selection of participants, classification of exposures, measurement of outcomes, and selection of reported results. Studies were categorised as low risk, moderate risk, serious risk, and critical risk of bias under each domain. Supplementary table 2 presents the results of the risk of bias assessment.

### Statistical methods

Odds ratios, relative risks, and hazard ratios (along with 95% confidence intervals) for comparison of the highest versus lowest categories of total, animal, and plant protein intake were used to calculate log odds ratios, relative risks, and hazard ratios with standard errors. A random effects model was used for analyses, in which we calculated both the Q statistic and I^2^ as indicators of heterogeneity.^24^ ^25^ ^26^ ^27^ I^2^ values greater than 50% were considered as significant heterogeneity between studies.^21^ A random effects model can account for variation between studies, and thus it can provide more conservative results than a fixed effects model.^28^ ^29^

For studies that reported effect sizes separately for intake of animal and plant protein, we first combined the estimates by using the fixed effects model to obtain an overall estimate and then included the pooled effect size in the meta-analysis. Studies that investigated only cancer or cardiovascular disease mortality in relation to protein intake were also considered in the meta-analysis of all cause mortality. If an estimate was reported for the lowest category of protein intake compared with the highest category, we computed the highest versus lowest estimates using the Orsini method.^30^ When significant heterogeneity between studies was found, we performed a subgroup analysis to examine possible sources of heterogeneity. These analyses were based on study location, duration of follow-up, sex, dietary assessment tools, health status of study participants, high versus low/middle income countries, single/repeated measurements of protein intake, effect size type, and statistical controlling for confounders (body mass index (BMI), total energy intake, and macronutrients (fat and carbohydrate)). Heterogeneity between subgroups was examined with a fixed effects model. 

Publication bias was examined by visual inspection of funnel plots. Formal statistical assessment of funnel plot asymmetry was also done with Egger’s regression asymmetry test and Begg’s test. A trim and fill method was used to detect the effect of probable missing studies on the overall effect. We also conducted a sensitivity analysis using a fixed effects model, in which each prospective cohort study was excluded in turn to examine the influence of that study on the overall estimate.

A method suggested by Greenland^31^ and Orsini^30^ was used to compute the trend from the odds ratios, relative risks, or hazard ratios estimates and their respective 95% confidence intervals across categories of protein intake. In this method, the distribution of cases and the odds ratios, relative risks, or hazard ratios with the variance estimates for three or more quantitative categories of exposure were required. We considered the midpoint of dietary protein intake in each category. For studies that reported the protein intake as a range, we estimated the midpoint in each category by calculating the mean of the lower and upper bound. When the highest and lowest categories were open ended, we assumed the length of these open ended intervals to be the same as those of the adjacent intervals. 

A two stage, random effects dose-response meta-analysis was applied to examine a possible non-linear association between protein intake and mortality. This meta-analysis was done through modelling of protein intake and restricted cubic splines with three knots at fixed centiles of 10%, 50%, and 90% of the distribution. Based on the Orsini method,^30^ we calculated restricted cubic spline models by a generalised least squares trend estimation method, which takes into account the correlation within each set of reported odds ratios, relative risks, or hazard ratios. The study specific estimates were then combined by the restricted maximum likelihood method in a multivariate random effects meta-analysis.^32^ A probability value for non-linearity was estimated by null hypothesis testing, in which the coefficient of the second spline was considered equal to zero. A linear dose-response association between an additional 3% of energy from proteins and mortality was investigated by use of the two stage generalised least squares trend estimation method. Study specific slope lines were first estimated and then these lines were combined to obtain an overall average slope.^30^ Study specific slope lines were combined by a random effects model. Statistical analyses were conducted using STATA version 14.0. A P value of less than 0.05 was considered significant for all tests, including Cochran’s Q test.

### Patient and public involvement

No patients were involved in setting the research question or the outcome measures, nor were they involved in developing plans for design, or implementation of the study. No patients were asked to advise on interpretation or writing up of results. There are no plans to disseminate the results of the research to study participants or the relevant patient community.

## Results

### Literature search

Overall, 18 683 articles were identified in the initial search. After exclusion of duplicate papers and those that did not meet the inclusion criteria, 57 full text articles of potentially relevant studies were identified. After full text review, an additional 25 articles were excluded: seven that enrolled patients with chronic renal diseases or who were undergoing haemodialysis, six that were conducted in the intensive care unit or on critically ill patients, one that was conducted on patients with end stage cancer, and four that reported associations with dietary sources of protein, rather than intake of total protein.^34^ ^35^ ^36^ ^37^ One article that combined mortality and ischaemic heart disease as the outcome was also excluded.^38^ Another paper that had considered urine urea nitrogen as a surrogate index of protein intake and reported the hazard ratio for mortality across categories of overnight urine urea nitrogen was excluded.^39^ One article that had considered total dietary patterns^40^ and three with insufficient data^41^ ^42^ ^43^ were also excluded. In one study, the type of protein intake was assessed rather than amount in relation to mortality and was therefore excluded.^44^

**Fig 1:**
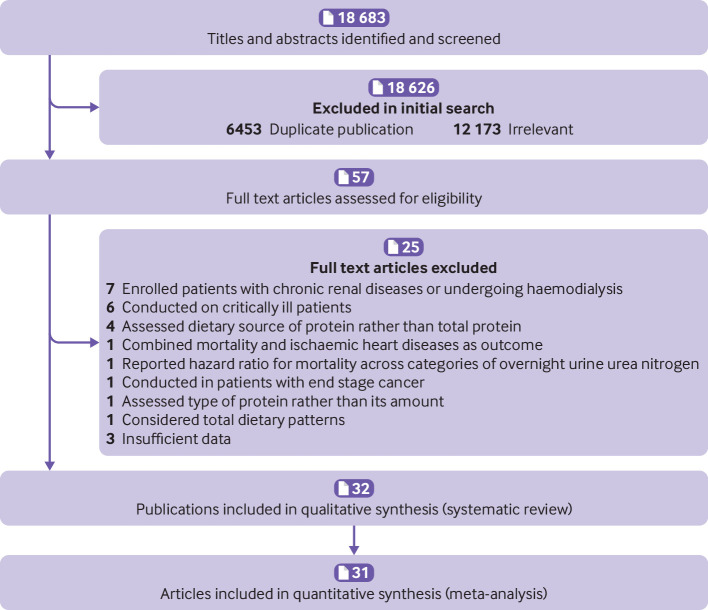
Flow diagram of study selection

Finally, 32 papers of cohort studies were included in the systematic review,^7^ ^9^ ^10^ ^11^ ^12^ ^13^ ^14^ ^15^ ^16^ ^17^ ^18^ ^45^ ^46^ ^47^ ^48^ ^49^ ^50^ ^51^ ^52^ ^53^ ^54^ ^55^ ^56^ ^57^ ^58^ ^59^ ^60^ ^61^ ^62^ ^63^ ^64^ ^65^ and 31 papers were included in this meta-analysis.^7^ ^9^ ^10^ ^11^ ^12^ ^13^ ^14^ ^15^ ^16^ ^17^ ^18^ ^45^ ^46^ ^47^ ^48^ ^49^ ^50^ ^51^ ^52^ ^53^ ^54^ ^55^ ^56^ ^57^ ^58^ ^59^ ^60^ ^61^ ^63^ ^64^ ^65^ Twenty two papers reported effect sizes for all cause mortality,^7^ ^9^ ^10^ ^11^ ^12^ ^13^ ^14^ ^15^ ^16^ ^17^ ^18^ ^46^ ^47^ ^49^ ^50^ ^51^ ^52^ ^54^ ^55^ ^59^ ^63^ ^65^ 17 for cardiovascular disease mortality,^9^ ^10^ ^11^ ^13^ ^14^ ^15^ ^16^ ^17^ ^18^ ^47^ ^49^ ^50^ ^51^ ^53^ ^57^ ^58^ ^64^ and 14 for cancer mortality.^7^ ^9^ ^13^ ^14^ ^15^ ^16^ ^17^ ^18^ ^45^ ^46^ ^48^ ^56^ ^60^ ^61^ Of these publications, 26 had reported effect sizes for intake of total protein,^7^ ^9^ ^10^ ^12^ ^14^ ^16^ ^17^ ^18^ ^45^ ^46^ ^47^ ^48^ ^49^ ^50^ ^51^ ^52^ ^53^ ^54^ ^55^ ^56^ ^59^ ^60^ ^61^ ^63^ ^64^ ^65^ 16 for intake of animal protein,^7^ ^9^ ^10^ ^12^ ^13^ ^14^ ^15^ ^16^ ^18^ ^45^ ^49^ ^56^ ^58^ ^61^ ^62^ ^64^ and 18 for intake of plant protein.^7^ ^9^ ^10^ ^11^ ^12^ ^13^ ^14^ ^15^ ^16^ ^18^ ^45^ ^49^ ^56^ ^57^ ^58^ ^61^ ^62^ ^64^ Figure 1 shows a flow diagram of study selection. 

Of 32 included publications in the systematic review, some studies were done on the same populations. The study by Song et al^15^ was conducted on the Nurses’ Health Study and the Health Professionals Follow-up Study datasets, and the study by Preis et al^64^ was conducted on the Health Professionals Follow-up Study dataset. In the current study, the study by Song et al^15^ was included in the main analysis because it was more complete than the study by Preis et al, but it lacked the required data for the dose-response analysis between intake of total protein and mortality. The study by Preis et al^64^ had reported such information, however, so was included in the dose-response analysis. The study by Song et al^15^ was considered in the calculation of total number of participants and cases of mortality. 

In addition, two papers (Papanikolaou et al 2019^13^ and Levine et al 2014^17^) were published based on the dataset of the Third National Health and Nutrition Examination Survey (NHANES III). The study by Papanikolaou et al^13^ was included in the main analysis owing to its comprehensiveness; however, because of lack of required data for the dose-response analysis in that study,^13^ we also used the study by Levine et al.^17^

Three additional studies ((Holmes et al 1999 and 2017,^55^ ^56^ Song et al 2018^7^) were also published, based on the Nurses’ Health Study or Health Professionals Follow-up Study datasets. All three studies were on patients with cancer, who were excluded from the other studies published from these datasets. Therefore these three studies were included. The two studies by Holmes et al^55^ ^56^ were performed on patients with breast cancer in the Nurses’ Health Study dataset; one had reported the effect size for cancer mortality and the other the effect size for all cause mortality. Therefore both were included. To calculate total number of participants and cases of mortality, one of the duplicate publications (Holmes et al 2017,^56^ Song et al 2016,^15^ Papanikolaou et al 2019^13^) was considered. 

### Characteristics of included studies

Tables 1-3 show the characteristics of the included prospective cohort studies. The number of participants in these studies ranged from 288 to 135 335, with an age range between 19 and 101 years. In total 715 128 participants were included in the 32 publications considered in this systematic review. During the follow-up periods ranging from 3.5 to 32 years, the total number of deaths from all causes was 113 039, from cardiovascular disease was 16 429,‬ and from cancer was 22 303. The sample size from the most comprehensive report was considered when it was published more than once.^13^ ^15^ ^56^ Three articles included only men,^12^ ^61^ ^64^ and seven publications included only women.^11^ ^14^ ^48^ ^55^ ^56^ ^60^ ^63^ Of the remaining studies, three papers had reported hazard ratios for men and women separately.^9^ ^52^ ^58^ In total, 14 publications described studies in the United States,^7^ ^11^ ^13^ ^14^ ^15^ ^17^ ^52^ ^53^ ^55^ ^56^ ^61^ ^62^ ^63^ ^64^ 17 in non-US countries,^9^ ^10^ ^12^ ^16^ ^18^ ^45^ ^46^ ^47^ ^48^ ^49^ ^50^ ^54^ ^57^ ^58^ ^59^ ^60^ ^65^ and 1 the populations from 18 different countries.^51^

**Table 1:** Characteristics of included studies for association between protein intake and all cause mortality in adults aged 19 or older

Author, country	Age*	Sample size	Follow-up (years)†	No of cases	Exposure	Exposure assessment	Health status‡	Comparison for protein intake	Effect size (95% CI)§	Adjustment¶
Virtanen 2019, Finland	42-60	M 2641	22.3	1225	Total protein	Food record	Healthy	>109.1 *v* <78.5 g/day	HR 1.17 (0.99 to 1.39)	1, 6, 7, 9, 13, 14, 15, 18, 20, 21, 22, 23, 49, 56, 62
					Animal protein			>82.1 *v* <49.2 g/day	HR 1.13 (0.95 to 1.35)	
					Plant protein			>32.2 *v* <19.6 g/day	HR 0.98 (0.76 to 1.26)	
Hernández-Alonso 2016, Spain	55-80	M 3071, W 4145	4.8	323	Total protein	FFQ	Unhealthy	≥18.54 *v* 15.9-17 %kcal	HR 1.66 (1.13 to 2.43)	1, 2, 9, 13, 14, 15, 18, 19, 22, 42, 46, 49, 56
					Animal protein			≥13 *v* 10.4-11.5 %kcal	HR 1.92 (1.31 to 2.82)	
					Plant protein			≥6.26 *v* 5.27-5.7 %kcal	HR 1.32 (0.88 to 2.00)	
Song 2016, US	49	M 46 329, W 85 013	26-32	36 115	Animal protein	FFQ	Healthy	>18 *v* ≤10 %kcal	HR 1.03 (0.98 to 1.08)	1, 9, 13, 15, 18, 31, 32, 38, 39, 40, 41, 48
					Plant protein			>6 *v* ≤3 %kcal	HR 0.89 (0.84 to 0.96)	
Zaslavsky 2017, US	65-84	W 10 034	12.4	3259	Total protein	FFQ	Healthy	>75 *v* <60 g/kg/day	HR 0.74 (0.54 to 1.01)	1, 6, 9, 13, 14, 15, 63
Dehghan 2017, 18 countries**	35-70	M 56 422, W 78 913	7.4	5796	Total protein	FFQ	Healthy	≥18.3 *v* ≤11.95 %kcal	HR 0.88 (0.77 to 1.00)	1, 5, 11, 13, 14, 15, 63
Courand 2016, France	45.1	M 653, F 475	10	289	Total protein	UUN	Unhealthy	>0.93 *v* <0.70 g/kg/day	HR 0.75 (0.56 to 0.99)	1, 2, 15, 25, 52, 54, 55, 58
Argos 2013, Bangladesh	36.9	M 6535, W 10 709	9	818	Total protein	FFQ	Healthy	≥12.7 *v* ≤11.4 %kcal	HR 1.07 (0.85 to 1.35)	1, 2, 9, 12, 14, 15, 19, 42, 44, 63
Kelemen 2005, US	55-69	W 29 017	15	3987	Total protein	FFQ	Healthy	≥20.7 *v* ≤15.2 %kcal	RR 0.99 (0.71 to 1.38)	1, 9, 13, 14, 15, 20, 21, 22, 23, 48, 51, 56, 63
					Animal protein			≥16.1 *v* ≤10.1 %kcal	RR 0.82 (0.59 to 1.13)	
					Plant protein			≥5.7 *v* ≤4 %kcal	RR 0.95 (0.82 to 1.10)	
Payette 1999, Canada	60-94	M 81, W 207	3.5	102	Total protein	Food recall	Healthy	≥0.8 *v* <0.8 g/kg/day	RR 1.20 (0.86 to 1.68)	Unadjusted
Papanikolaou 2019, US	19-99	Both 17 199	18	4280	Animal protein	Food recall	Healthy	NR	HR 1.01 (0.99 to 1.02)	Unclear
					Plant protein				HR 0.99 (0.97 to 1.01)	
Sun 2019, US	50-79	W 127 495	24	35 043	Plant protein	FFQ	Healthy	NR	HR 0.91 (0.86 to 0.95)	Unclear
Levine 2014, US	≥50	M 2846, W 3535	18	2578	Total protein	Food recall	Healthy	≥20 *v* ≤10 %kcal	HR 0.93 (0.74 to 1.19)	1, 2, 3, 4, 15, 56, 14, 63
Budhathoki 2019, Japan	40-69	M 32 201, W 38 495	18	12 381	Total protein	FFQ	Healthy	≥17 *v* ≤11.82 %kcal	HR 0.99 (0.90 to 1.09)	1, 6, 9, 13, 14, 15, 16, 17, 18, 20, 21, 22, 23, 31, 32
					Animal protein			≥10.65 *v* ≤4.95 %kcal	HR 0.98 (0.88 to 1.08)	
					Plant protein			≥8.1 *v* ≤5.32 %kcal	HR 0.87 (0.78 to 0.96)	
Halbesma 2009, Netherlands	20-75	Both 8461	6.4	443	Total protein	UUN	Healthy	≥1.4 *v* 1.11-1.25 g/kg/day	HR 1.03 (0.73 to 1.43)	1, 2, 59
Holmes 1999, US	54	W 1982	13	378	Total protein	FFQ	Unhealthy	≥81.5 *v* ≤60.9 g/day	RR 0.65 (0.47 to 0.88)	1, 8, 9, 14, 50, 51, 35, 48, 61
Mendonça 2019, UK	≥85	M 289, W 428	5	457	Total protein	Food recall	Healthy	≥0.8 *v* ˂0.8 g/kg/day	HR 0.91 (0.73 to 1.14)	1, 2, 14, 56, 63
Chan 2019, China	≥65	M 1480	13.8	594	Total protein	FFQ	Healthy	≥20 *v* ≤12.85 %kcal	HR 0.77 (0.58 to 1.01)	1, 7, 9, 14, 15, 18, 37, 39, 40, 41, 56
					Animal protein			≥12.8 *v* ≤5.85 %kcal	HR 0.79 (0.60 to 1.03)	
					Plant protein			≥9.3 *v* ≤5.1 %kcal	HR 1.08 (0.82 to 1.43)	
Chan 2019, China	≥65	W 1540	13.8	369	Total protein	FFQ	Healthy	≥19.8 *v* ≤12.85 %kcal	HR 0.67 (0.46 to 0.96)	1, 7, 9, 14, 15, 18, 37, 39, 40, 41, 56
					Animal protein			≥11.9 *v* ≤5.3 %kcal	HR 0.84 (0.58 to 1.20)	
					Plant protein			≥9.9 *v* ≤5.8 %kcal	HR 0.65 (0.45 to 0.92)	
Dwyer 1994, US	65-74	W 1341	14.5	48	Total protein	Food recall	Healthy	Per 15 g increase	RR 1.04 (0.96 to 1.11)	1
Dwyer 1994, US	65-74	M 1231	14.5	71	Total protein	Food recall	Healthy	Per 15 g increase	RR 1.00 (0.95 to 1.06)	1
Song 2018, US	30-75	M 623, W 919	9	817	Total protein	FFQ	Unhealthy	Per 3.2 % increase	HR 1.10 (1.03 to 1.70)	1, 2, 9, 13, 14, 15, 18, 31, 32, 41, 49, 60
					Animal protein			Per 3.4 % increase	HR 1.12 (1.04 to 1.20)	
					Plant protein			Per 1.4 % increase	HR 0.94 (0.85 to 1.03)	
Bates 2010, UK	≥65	M 548, F 552	13	749	Total protein	Food record		M per 17 g increase, F per 14 g increase	HR 0.86 (0.77 to 0.97)	1, 2
Campmans-Kuijpers 2015, European countries	57.5	M 2255, W 1827	9.4	787	Total protein	FFQ	Unhealthy	Per 5% increase	HR 1.00 (0.88 to 1.10)	1, 9, 14, 15, 18, 30, 41, 45, 49, 63
					Animal protein			Per 5% increase	HR 1.01 (0.90 to 1.10)	
					Plant protein			Per 5% increase	HR 0.55 (0.32 to 0.90)	
Tharrey 2018, US-Canada	≥25	Both 81 377	9.4	2276	Animal protein	FFQ	Healthy	Per 18 g increase	HR 1.12 (1.05 to 1.19)	1, 2, 3, 6, 7, 9, 13, 15, 18, 20, 22, 25, 33, 36, 47, 63
					Plant protein			Per 18 g increase	HR 0.95 (0.89 to 1.02)	

1 kcal=4.18 kJ=0.00418 MJ.

FFQ=food frequency questionnaire; HR=hazard ratio; M=men; NR=not reported; OR=odds ratio; RR=risk ratio, UUN=urine urea nitrogen; W=women.

* Presented as mean or range.

† Number of years that individuals were followed up in the prospective cohort studies.

‡ People with comorbidities were considered as unhealthy.

§ These effect sizes are for comparison of the highest and the lowest categories.

¶ Adjustments: age (1), sex (2), race (3), waist circumference (4), urban or rural location (5), occupation status (6), marital status (7), menopausal status (8), body mass index (9), weight change (10), waist to hip ratio (11), height (12), physical activity (13), total energy (14), smoking (15), intake of green tea (16), coffee (17), alcohol (18), total fat (19), saturated fat (20), monounsaturated fat (21), polyunsaturated fat (22), trans fat (23), cholesterol (24), sodium (25), potassium (26), animal fat (27), vegetable fat (28), methionine (29), total protein (30), animal protein (31), plant protein (32), folate (33), magnesium (34), calcium (35) vitamin A, C, E, B6, and B12 (36), total grains (37), whole grains (38), fruit (39), vegetable (40), fibre (41), carbohydrates (42) amino acids (43), water arsenic concentration (44), healthy diet (45), glycaemic index (46), type of diet in the vegetarian spectrum (47), use of supplements (48), drug treatment (49), oral contraceptive (50), postmenopausal hormone (51), serum lipids (52), glucose intolerance (53), systolic blood pressure (54), estimated glomerular filtration rate (55), chronic diseases at baseline and follow-up (56), number of frailty criteria (57), fasting glucose (58), cardiovascular risk factors (59), cancer stage (60), tumour size (61), duration of diabetes (62), education (63), social class (64).

** Canada, Sweden, United Arab Emirates, Argentina, Brazil, Chile, China, Colombia, Iran, Malaysia, occupied Palestinian territory, Poland, South Africa, Turkey, Bangladesh, India, Pakistan, and Zimbabwe.

**Table 2:** Characteristics of included studies for the association between protein intake and cardiovascular disease mortality in adults aged over 18

Author, country	Age*	Sample size	Follow-up (years)†	No of cases	Exposure	Exposure assessment	Health status‡	Comparison for protein intake	Effect size (95% CI)§	Adjustment¶
Hernández-Alonso 2016, Spain	55-80	M 3071, W 4145	4.8	81	Total protein	FFQ	Unhealthy	≥18.54 *v* 15.9-17 %kcal	HR 2.10 (0.93 to 4.75)	1, 2, 9, 13, 14, 15, 18, 19, 22, 42, 46, 49, 56
					Animal protein			≥13 *v* 10.4-11.5 %kcal	HR 2.98 (1.36 to 6.51)	
					Plant protein			≥6.26 *v* 5.27-5.7 %kcal	HR 0.78 (0.32 to 1.88)	
Song 2016, US	49	M 46 329, W 85 013	26-32	8851	Animal protein	FFQ	Healthy	>18 *v* ≤10 %kcal	HR 1.09 (0.99 to 1.20)	1, 9, 13, 15, 18, 31, 32, 38, 39, 40, 41, 48
					Plant protein			>6 *v* ≤3 %kcal	HR 0.88 (0.80 to 0.97)	
Dehghan 2017, 18 countries**	35-70	M 56 422, W 78 913	7.4	1649	Total protein	FFQ	Healthy	≥18.3 *v* ≤11.95 %kcal	HR 0.90 (0.71 to 1.15)	1, 5, 11, 13, 14, 15, 63
Courand 2016, France	45.1	M 653, W 475	10	202	Total protein	FFQ	Unhealthy	>0.93 *v* <0.70 g/kg/day	HR 0.76 (0.54 to 1.06)	1, 2, 15, 25, 52, 54, 58
Kelemen 2005, US	55-69	W 29 017	15	739	Total protein	FFQ	Healthy	≥20.7 *v* ≤15.2 %kcal	RR 0.84 (0.39 to 1.79)	1, 9, 13, 14, 15, 20, 21, 22, 23, 48, 51, 56, 63
					Animal protein			≥16.1 *v* ≤10.1 %kcal	RR 0.88 (0.42 to 1.86)	
					Plant protein			≥5.7 *v* ≤4 %kcal	RR 0.70 (0.49 to 0.99)	
Sauvaget 2004, Japan	35-89	M 1436, W 2295	14	60	Total protein	Food record	Healthy	≥79.5 *v* ≤57.5 g/day	HR 0.42 (0.20 to 0.85)	1, 2, 9, 15, 18, 56, 60
					Animal protein			≥43.5 *v* ≤25.5 g/day	HR 0.45 (0.23 to 0.89)	
					Plant protein			≥39.5 *v* ≤28.5 g/day	HR 1.12 (0.57 to 2.21)	
Nagata 2015, Japan	35-101	M 13 355	16	328	Total protein	FFQ	Healthy	NR	HR 1.01 (0.73 to 1.40)	1, 7, 9, 13, 14, 15, 18, 20, 21, 22, 23, 25, 30, 41
					Animal protein				HR 1.01 (0.74 to 1.38)	
					Plant protein				HR 1.14 (0.72 to1.80)	
Nagata 2015, Japan	35-101	W 15 724	16	349	Total protein	FFQ	Healthy	NR	HR 1.01 (0.74 to 1.38)	1, 7, 8, 9, 13, 14, 15, 18, 20, 21, 22, 23, 25, 30, 41
					Animal protein				HR 1.26 (0.81 to 1.96)	
					Plant protein				HR 0.81 (0.52 to 1.26)	
Papanikolaou 2019, US	19-99	Both 17 199	18	-	Animal protein	Food recall	Healthy	NR	HR 1.01 (0.99 to 1.02)	Unclear
					Plant protein				HR 1.00 (0.97 to 1.02)	
Sun 2019, US	50-79	W 127 495	24	-	Total protein	FFQ	Healthy	NR	HR 0.87 (0.79 to 0.95)	Unclear
Kurihara 2019, Japan**]**	≥30	M 3224, W 4520	13.9	354	Plant protein	Food record	Healthy	≥7.9 *v* ≤6.6 %kcal	HR 0.86 (0.75 to 0.99)	1, 2, 9, 15, 18, 25, 26, 27, 31, 41
Levine 2014, US	≥50	M 2846, W 3535	18	1193	Total protein	Food recall	Healthy	≥20 *v* ≤10 %kcal	HR 0.88 (0.63 to 1.22)	1, 2, 3, 4, 15, 56, 14, 63
Budhathoki 2019, Japan	40-69	M 32 201, W 38 495	18	3025	Total protein	FFQ	Healthy	≥17 *v* ≤11.82 %kcal	HR 0.97 (0.80 to 1.18)	1, 6, 9, 13, 14, 15, 16, 17, 18, 20, 21, 22, 23, 31, 32
					Animal protein			≥10.65 *v* ≤4.95 %kcal	HR 0.97 (0.79 to 1.19)	
					Plant protein			≥8.1 *v* ≤5.32 %kcal	HR 0.73 (0.59 to 0.91)	
Preis 2010, US	40-75	M 43 960	18	1155	Total protein	FFQ	Healthy	≥21.15 *v* ≤15.65 %kcal	RR 1.05 (0.85 to 1.30)	1, 9, 13, 14, 15, 18, 20, 21, 22, 23, 33, 34, 41, 46, 48, 56
					Animal protein			≥16.3 *v* ≤10.4 %kcal	RR 1.10 (0.88 to 1.37)	
					Plant protein			≥6 *v* ≤4 %kcal	RR 0.66 (0.49 to 0.88)	
Chan 2019, China	≥65	M 1480	13.8	117	Total protein	FFQ	Healthy	≥20 *v* ≤12.85 %kcal	HR 0.75 (0.41 to 1.39)	1, 7, 9, 14, 15, 18, 37, 39, 40, 41, 56
					Animal protein			≥12.8 *v* ≤5.85 %kcal	HR 0.75 (0.41 to 1.39)	
					Plant protein			≥9.3 *v* ≤5.1 %kcal	HR 0.92 (0.47 to 1.77)	
Chan 2019, China	≥65	W 1540	13.8	117	Total protein	FFQ	Healthy	≥19.8 *v* ≤12.85 %kcal	HR 0.78 (0.38 to 1.62)	1, 7, 9, 14, 15, 18, 37, 39, 40, 41, 56
					Animal protein			≥11.9 *v* ≤5.3 %kcal	HR 0.93 (0.42 to 2.03)	
					Plant protein			≥9.9 *v* ≤5.8 %kcal	HR 0.71 (0.35 to 1.45)	
Bates 2010, UK	≥65	M 548, W 552	13	199	Total protein	Food record	Healthy	M per 17 g increase, F per 14 g increase	HR 0.79 (0.67 to 0.94)	1, 2
Campans-Kuijpers 2015, European countries	57.5	M 2255, W 1827	9.4	266	Total protein	FFQ	Unhealthy	Per 5% increase	HR 1.00 (0.82 to 1.23)	1, 9, 14, 15, 18, 30, 41, 45, 49, 63
					Animal protein			Per 5% increase	HR 1.02 (0.83 to 1.25)	
					Plant protein			Per 5% increase	HR 0.81 (0.33 to 1.99)	
Esrey 1996, US	30-59	M 2071, W 1854	12	52	Total protein	Food recall	Healthy	Per 1% increase	RR 1.01 (0.95 to 1.07)	1, 2, 9, 15, 52, 53, 54
Esrey 1996, US	60-79	M 282, W 339	12	40	Total protein	Food recall	Healthy	Per 1% increase	RR 1.00 (0.93 to 1.08)	1, 2, 9, 15, 52, 53, 54

1 kcal=4.18 kJ=0.00418 MJ.

FFQ=food frequency questionnaire; HR=hazard ratio; M=men; NR=not reported; OR=odds ratio; RR=risk ratio, UUN=urine urea nitrogen; W=women.

* Presented as mean or range.

† Number of years that individuals were followed up in the prospective cohort studies.

‡ People with comorbidities were considered as unhealthy.

§ These effect sizes are for comparison of the highest and the lowest categories.

¶ Adjustments: age (1), sex (2), race (3), waist circumference (4), urban or rural location (5), occupation status (6), marital status (7), menopausal status (8), BMI (9), weight change (10), waist to hip ratio (11), height (12), physical activity (13), total energy (14), smoking (15), intake of green tea (16), coffee (17), alcohol (18), total fat (19), saturated fat (20), monounsaturated fat (21), polyunsaturated fat (22), trans fat (23), cholesterol (24), sodium (25), potassium (26), animal fat (27), vegetable fat (28), methionine (29), total protein (30), animal protein (31), plant protein (32), folate (33), magnesium (34), calcium (35) vitamin A, C, E, B6, and B12 (36), total grains (37), whole grains (38), fruit (39), vegetable (40), fibre (41), carbohydrates (42) amino acids (43), water arsenic concentration (44), healthy diet (45), glycaemic index (46), type of diet in the vegetarian spectrum (47), use of supplements (48), drug treatment (49), oral contraceptive (50), postmenopausal hormone (51), serum lipids (52), glucose intolerance (53), systolic blood pressure (54), estimated glomerular filtration rate (55), chronic diseases at baseline and follow-up (56), number of frailty criteria (57), fasting glucose (58), cardiovascular risk factors (59), cancer stage (60), tumour size (61), duration of diabetes (62), education (63), social class (64).

** Canada, Sweden, United Arab Emirates, Argentina, Brazil, Chile, China, Colombia, Iran, Malaysia, occupied Palestinian territory, Poland, South Africa, Turkey, Bangladesh, India, Pakistan, and Zimbabwe.

**Table 3:** Characteristics of included studies for the association between protein intake and cancer mortality in adults aged >18 years

Author, country	Age*	Sample size	Follow-up (years)†	No of cases	Exposure	Exposure assessment	Health status‡	Comparison for protein intake	Effect size (95% CI)§	Adjustment¶
Hernández-Alonso 2015, Spain	55-80	M 3071, W 4145	4.8	130	Total protein	FFQ	Unhealthy	≥18.54 *v* 15.9-17 %kcal	HR 1.44 (0.80 to 2.59)	1, 2, 9, 13, 14, 15, 18, 19, 22, 42, 46, 49, 56
					Animal protein			≥13 *v* 10.4-11.5 %kcal	HR 1.81 (1.00 to 3.31)	
					Plant protein			≥6.26 *v* 5.27-5.7 %kcal	HR 1.39 (0.70 to 2.75)	
Song 2016, US	49	M 46 329, W 85 013	26-32	13 159	Animal protein	FFQ	Healthy	>18 *v* ≤10 %kcal	HR 1.02 (0.94 to 1.11)	1, 9, 13, 15, 18, 31, 32, 38, 39, 40, 41, 48
					Plant protein			>6 *v* ≤3 %kcal	HR 0.92 (0.82 to 1.03)	
Argos 2013, Bangladesh	36.9	M 6535, W 10 709	9	135	Total protein	FFQ	Healthy	≥12.7 *v* ≤11.4 %kcal	HR 1.79 (0.99 to 3.25)	1, 2, 9, 12, 14, 15, 19, 42, 44, 63
Kelemen 2005, US	55-69	W 29 017	15	1676	Total protein	FFQ	Healthy	≥20.7 *v* ≤15.2 %kcal	RR 1.07 (0.64 to 1.79)	1, 9, 13, 14, 15, 20, 21, 22, 23, 48, 51, 56, 63
					Animal protein			≥16.1 *v* ≤10.1 %kcal	RR 0.77 (0.47 to 1.27)	
					Plant protein			≥5.7 *v* ≤4 %kcal	RR 1.04 (0.83 to 1.32)	
Smit 2007, US	35-79	M 9777	12	167	Total protein	Food recall	Healthy	≥104 *v* ≤61 g/day	OR 1.32 (0.81 to 2.17)	1, 5, 9, 13, 14, 15, 63
					Animal protein			≥41 *v* ≤13 g/day	OR 1.01 (0.52 to 1.96)	
					Plant protein			≥32 *v* ≤16 g/day	OR 1.19 (0.66 to 2.13)	
Papanikolaou 2019, US	19-99	Both 17 199	18	-	Animal protein	Food recall	Healthy	NR	HR 1.00 (0.98 to 1.03)	Unclear
					Plant protein				HR 1.01 (0.97 to 1.05)	
Levine 2014, US	≥50	M 2846, W 3535	18	630	Total protein	Food recall	Healthy	≥20 *v* ≤10 %kcal	HR 0.89 (0.56 to 1.44)	1, 2, 3, 4, 15, 56, 14, 63
Budhathoki 2019, Japan	40-69	M 32 201, W 38 495	18	5055	Total protein	FFQ	Healthy	≥17 *v* ≤11.82 %kcal	HR 1.00 (0.86 to 1.16)	1, 6, 9, 13, 14, 15, 16, 17, 18, 20, 21, 22, 23, 31, 32
					Animal protein			≥10.65 *v* ≤4.95 %kcal	HR 0.97 (0.83 to 1.14)	
					Plant protein			≥8.1 *v* ≤5.32 %kcal	HR 1.04 (0.88 to 1.23)	
Holmes 2017, US	30-55	W 6348	29	919	Total protein	FFQ	Unhealthy	≥84.6 *v* ≤66.7 g/day	RR 0.95 (0.77 to 1.17)	1, 8, 9, 13, 14, 15, 10, 18, 49, 50, 51, 59, 60
					Animal protein			≥64.6 *v* ≤46.8 g/day	RR 0.85 (0.68 to 1.05)	
					Plant protein			≥23.3 *v* ≤16 g/day	RR 1.09 (0.87 to 1.37)	
Borugian 2004, Canada	19-75	W 603	10	112	Total protein	FFQ	Unhealthy	≥83 *v* ≤52 g/day	RR 0.40 (0.20 to 0.80)	1, 14, 59
Rohan 1993, Australia	20-74	W 412	5.5	112	Total protein	FFQ	Unhealthy	≥103 *v* ≤59 g/day	HR 0.74 (0.34 to 1.66)	1, 9, 14
Chan 2019, China	≥65<	M 1480	13.8	216	Total protein	FFQ	Healthy	≥20 *v* ≤12.85 %kcal	HR 0.66 (0.41 to 1.07)	1, 7, 9, 14, 15, 18, 37, 39, 40, 41, 56
					Animal protein			≥12.8 *v* ≤5.85 %kcal	HR 0.70 (0.44 to 1.12)	
					Plant protein			≥9.3 *v* ≤5.1 %kcal	HR 1.27 (0.80 to 2.03)	
Chan 2019, China	≥65	W 1540	13.8	120	Total protein	FFQ	Healthy	≥19.8 *v* ≤12.85 %kcal	HR 0.59 (0.31 to 1.13)	1, 7, 9, 14, 15, 18, 37, 39, 40, 41, 56
					Animal protein			≥11.9 *v* ≤5.3 %kcal	HR 0.76 (0.41 to 1.42)	
					Plant protein			≥9.9 *v* ≤5.8 %kcal	HR 0.57 (0.31 to 1.02)	
Song 2018, US	30-75	M 623, W 919	9	185	Total protein	FFQ	Unhealthy	Per 3.2% increase	HR 1.07 (0.94 to 1.23)	1, 2, 9, 13, 14, 15, 18, 31, 32, 41, 49, 60
					Animal protein			Per 3.4% increase	HR 1.13 (0.98 to 1.30)	
					Plant protein			Per 1.4% increase	HR 0.77 (0.62 to 0.95)	
Palli 2000, Italy	NR	M 239 W 143	11	317	Total protein	FFQ	Unhealthy	Unclear (T3 *v* T1)	HR 1.00 (0.75 to 1.32)	1, 2, 14, 64
					Animal protein			Unclear (T3 *v* T1)	HR 1.09 (0.83 to 1.44)	
					Plant protein			Unclear (T3 *v* T1)	HR 0.82 (0.62 to 1.09)	

1 kcal=4.18 kJ=0.00418 MJ.

FFQ=food frequency questionnaire; HR=hazard ratio; M=male; NR=not reported; OR=odds ratio; RR=risk ratio, UUN=urine urea nitrogen; W=women.

* Presented as mean or range.

† Number of years that individuals were followed up in the prospective cohort studies.

‡ People with comorbidities were considered as unhealthy.

§ These effect sizes are for comparison of the highest and the lowest categories.

¶ Adjustments: age (1), sex (2), race (3), waist circumference (4), urban or rural location (5), occupation status (6), marital status (7), menopausal status (8), BMI (9), weight change (10), waist to hip ratio (11), height (12), physical activity (13), total energy (14), smoking (15), intake of green tea (16), coffee (17), alcohol (18), total fat (19), saturated fat (20), monounsaturated fat (21), polyunsaturated fat (22), trans fat (23), cholesterol (24), sodium (25), potassium (26), animal fat (27), vegetable fat (28), methionine (29), total protein (30), animal protein (31), plant protein (32), folate (33), magnesium (34), calcium (35) vitamin A, C, E, B6, and B12 (36), total grains (37), whole grains (38), fruit (39), vegetable (40), fibre (41), carbohydrates (42) amino acids (43), water arsenic concentration (44), healthy diet (45), glycaemic index (46), type of diet in the vegetarian spectrum (47), use of supplements (48), drug treatment (49), oral contraceptive (50), postmenopausal hormone (51), serum lipids (52), glucose intolerance (53), systolic blood pressure (54), estimated glomerular filtration rate (55), chronic diseases at baseline and follow-up (56), number of frailty criteria (57), fasting glucose (58), cardiovascular risk factors (59), cancer stage (60), tumour size (61), duration of diabetes (62), education (63), social class (64).

§ Canada, Sweden, United Arab Emirates, Argentina, Brazil, Chile, China, Colombia, Iran, Malaysia, occupied Palestinian territory, Poland, South Africa, Turkey, Bangladesh, India, Pakistan, and Zimbabwe.

To examine protein intake, 11 publications had used dietary records or recalls^10^ ^12^ ^13^ ^17^ ^47^ ^52^ ^53^ ^57^ ^59^ ^61^ ^65^ and 19 had used a food frequency questionnaire.^7^ ^9^ ^11^ ^14^ ^15^ ^16^ ^18^ ^45^ ^46^ ^48^ ^49^ ^51^ ^55^ ^56^ ^58^ ^60^ ^62^ ^63^ ^64^ In the studies by Halbesma et al^54^ and Courand et al,^50^ intake of total protein was estimated with the use of overnight urine urea nitrogen. In total, 31 publications used baseline data of protein intake in their analysis (single measurement), whereas one article considered the average protein intake throughout the follow-up (repeated measurements) as the main exposure.^15^ All studies except for one^60^ adjusted the associations for age. 

Most cohorts controlled for some conventional risk factors, including BMI (n=24), smoking (n=22), and alcohol consumption (n=14). Others also adjusted for physical activity (n=14), energy intake (n=25), other dietary variables (n=14), and macronutrients (fat or carbohydrate; n=12). Based on the ROBINS-E tool, 15 articles had a low risk of bias in all components (supplementary table 2).^7^ ^11^ ^12^ ^13^ ^14^ ^15^ ^16^ ^17^ ^18^ ^46^ ^51^ ^55^ ^62^ ^63^ ^64^ Nine papers provided effect sizes for mortality from cardiovascular disease and cancer, without reporting any effect size for all cause mortality.^10^ ^45^ ^48^ ^53^ ^56^ ^57^ ^58^ ^60^ ^61^ The reported effect sizes in these studies were combined and the overall effect size was considered in the meta-analysis of all cause mortality.

### Systematic review

Of 29 articles on the association between intake of total protein and all cause mortality, six reported an inverse association,^9 10 47 48 54 55 ^one showed a positive association,^7^ and the others reported no significant association.^12-18 45 46 49-53 56 58-61 63-65 ^For the association between intake of animal protein and all cause mortality, two studies showed an inverse association^10 16^ and the others indicated no significant association.^7 9 12-15 18 45 49 58 61 62 64^ Moreover, seven publications showed an inverse association between intake of plant protein and all cause mortality.^9 11 16 18 49 57 64^ For cardiovascular disease mortality, two studies reported a protective association with intake of total protein,^10 47 ^one study with animal protein,^10^ and six articles with plant protein.^11 14 15 18 57 64^ One study indicated an inverse association between intake of total protein and cancer mortality.^48^ One study also showed an inverse association between intake of plant protein and cancer mortality.^7^

### Meta-analysis on protein intake and all cause mortality

**Fig 2:**
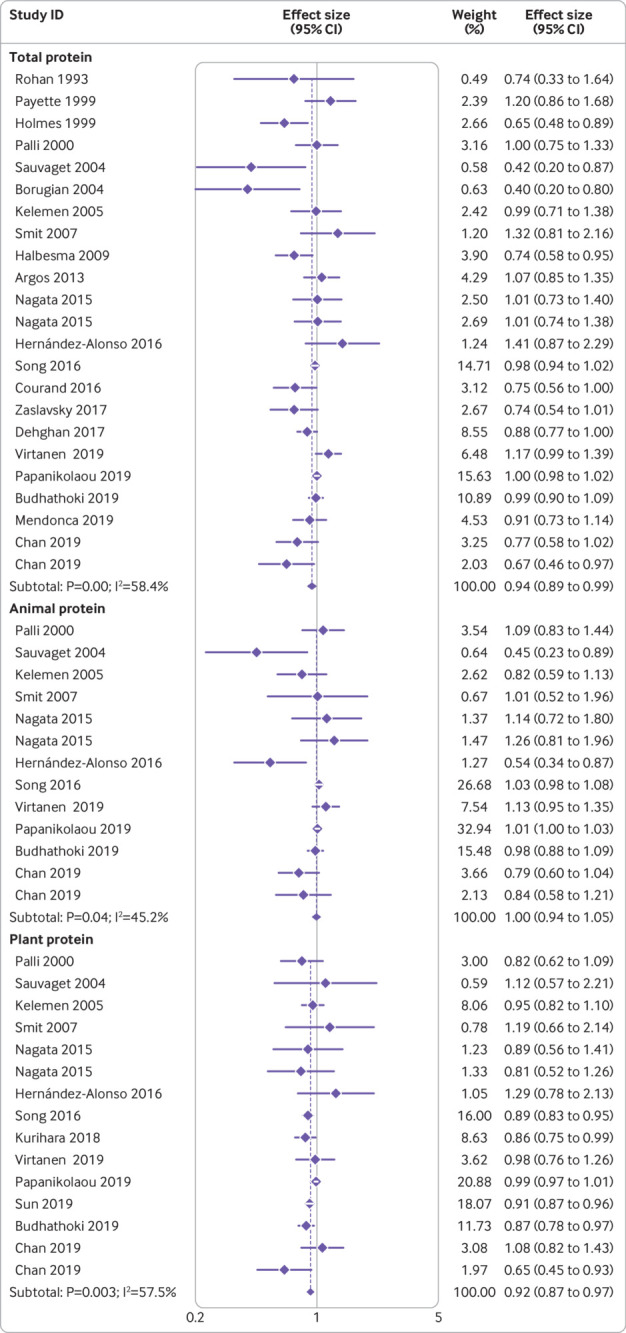
Forest plot for association between protein intake and risk of all cause mortality in adults aged 19 or older, expressed as comparison between highest and lowest categories of protein intake. Diamonds represent pooled estimates from random effects analysis

Of 29 papers on intake of total protein and all cause mortality, 21 presented sufficient data for comparison of the highest versus lowest categories of total protein intake.^9^ ^10^ ^12^ ^13^ ^14^ ^15^ ^16^ ^18^ ^45^ ^46^ ^48^ ^50^ ^51^ ^54^ ^55^ ^58^ ^59^ ^60^ ^61^ ^63^ ^65^ Of 480 304 participants included in these articles, 72 261 died. The summary effect size for all cause mortality comparing the highest and lowest intakes of total protein was 0.94 (95% confidence interval 0.89 to 0.99, P=0.02), indicating a significant inverse association between total protein intake and all cause mortality (fig 2). Significant heterogeneity was seen between studies (I^2^=58.4%, P<0.001).

When the association between consumption of animal protein and all cause mortality was examined in 11 publications,^10^ ^11^ ^13^ ^14^ ^15^ ^16^ ^17^ ^19^ ^56^ ^58^ ^61^ including a total of 304 100 participants and 60 495 deaths, no significant association was found (pooled effect size comparing highest and lowest intakes was 1.00, 95% confidence interval 0.94 to 1.05, P=0.86), with moderate heterogeneity among the studies (I^2^=45.2%, P=0.04; fig 2). Consumption of plant protein, however, which was examined in 13 articles^10^ ^11^ ^12^ ^13^ ^14^ ^15^ ^16^ ^17^ ^19^ ^56^ ^57^ ^58^ ^61^ with a total of 439 339 participants and 95 892 deaths, was inversely associated with all cause mortality (pooled effect size comparing the highest and lowest intakes was 0.92, 0.87 to 0.97, P=0.002), with significant heterogeneity among the studies (I^2^=57.5%, P=0.003; fig 2).

### Meta-analysis on protein intake and cardiovascular disease mortality

**Fig 3:**
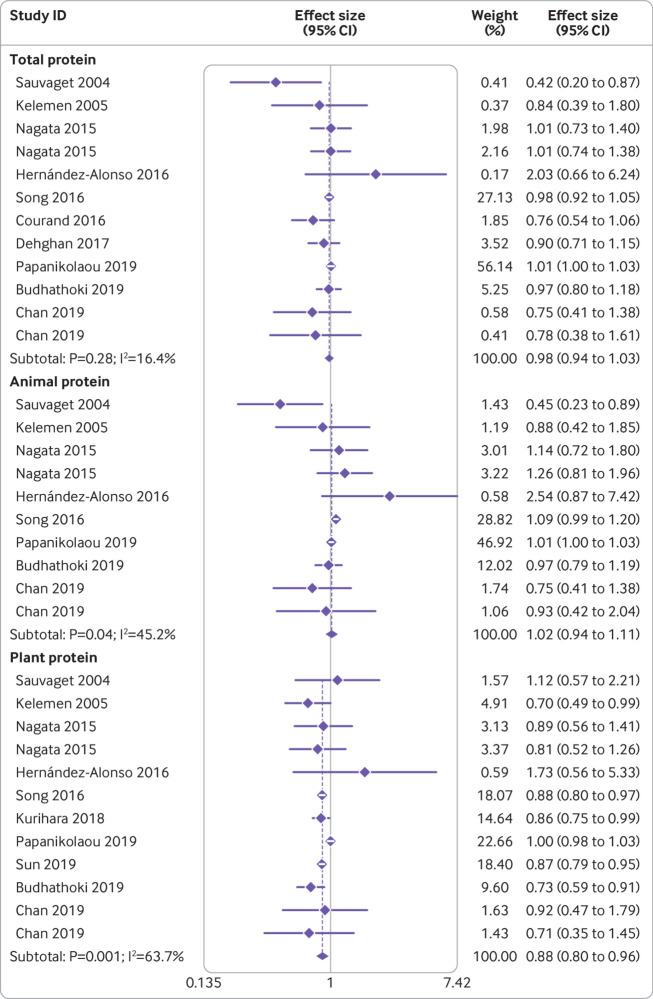
Forest plot for association between protein intake and risk of cardiovascular disease mortality in adults aged 19 or older, expressed as comparison between highest and lowest categories of protein intake. Diamonds represent pooled estimates from random effects analysis

Ten publications^9^ ^10^ ^13^ ^14^ ^15^ ^16^ ^18^ ^50^ ^51^ ^58^ examined the association between intake of total protein and risk of cardiovascular disease mortality. These studies included a total of 427 005 participants and 15 518 deaths. The summary effect size for cardiovascular disease mortality, comparing the highest and lowest protein intakes, was 0.98 (95% confidence interval 0.94 to 1.03, P=0.51), indicating no clear significant association between total protein intake and cardiovascular disease mortality (fig 3). No significant heterogeneity was seen among the studies (I^2^=16.4%, P=0.28).

The association between consumption of animal protein and cardiovascular disease mortality was examined in eight papers,^9^ ^10^ ^13^ ^14^ ^15^ ^16^ ^18^ ^58^ which included 290 542 participants and 13 667 deaths. No significant association was found (pooled effect size comparing the highest and lowest intakes was 1.02, 95% confidence interval 0.94 to 1.11, P=0.56), with no significant heterogeneity among the studies (I^2^=31.7%, P=0.16; fig 3). For plant protein consumption, however, which was examined in 10 articles^9^ ^10^ ^11^ ^13^ ^14^ ^15^ ^16^ ^18^ ^57^ ^58^ with a total of 425 781 participants and 14 021 deaths, an inverse association was found with cardiovascular disease (pooled effect size comparing the highest and lowest intakes was 0.88, 0.80 to 0.96, P=0.003; fig 3). No significant heterogeneity was found between studies (I^2^=63.7%, P=0.001).

### Meta-analysis on protein intake and cancer mortality

**Fig 4:**
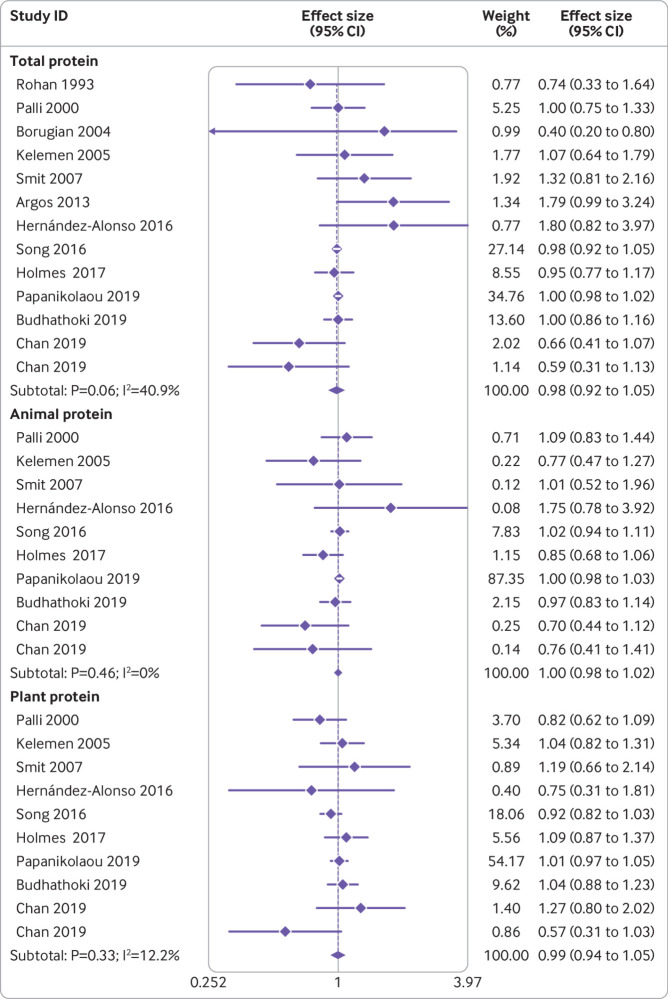
Forest plot for association between protein intake and risk of cancer mortality in adults aged 19 or older, expressed as comparison between highest and lowest categories of protein intake. Diamonds represent pooled estimates from random effects analysis

Twelve papers,^9^ ^13^ ^14^ ^15^ ^16^ ^18^ ^45^ ^46^ ^48^ ^56^ ^60^ ^61^ with a total of 292 629 participants and 22 118 deaths, examined the association between intake of total protein and cancer mortality. The summary effect size for cancer mortality comparing the highest and lowest protein intakes was 0.98 (95% confidence interval 0.92 to 1.05, P=0.63), indicating no clear association; however, evidence of moderate heterogeneity was found between studies (I^2^=40.9%, P=0.06; fig 4). The same findings were obtained for animal protein consumption and cancer mortality based on nine publications^9^ ^13^ ^14^ ^15^ ^16^ ^18^ ^45^ ^56^ ^61^ with a total of 274 370 participants and 21 759 deaths (pooled effect size comparing the highest and lowest protein intakes was 1.00, 95% confidence interval 0.98 to 1.02, P=0.88), with no significant heterogeneity among the studies (I^2^=0%, P=0.46; fig 4). This was also the case for plant protein consumption, which was examined in nine articles^9^ ^13^ ^14^ ^15^ ^16^ ^18^ ^45^ ^56^ ^61^ with a total of 274 370 participants and 21 759 deaths (pooled effect size comparing the highest and lowest protein intakes was 0.99, 0.94 to 1.05, P=0.68). Moreover, no significant heterogeneity among the studies was found in this case (I^2^=12.2%; P=0.33; fig 4).

### Linear and non-linear dose-response analysis

**Fig 5:**
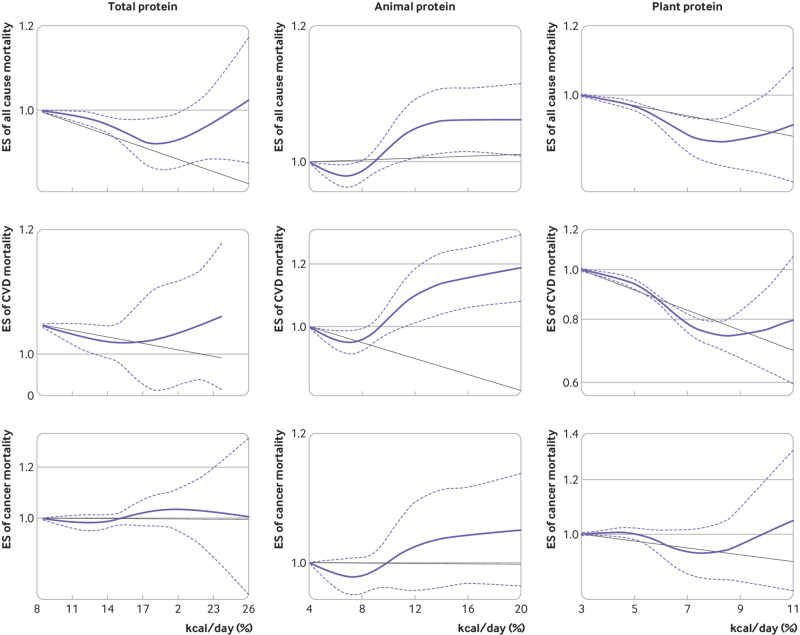
Non-linear dose-response association of intakes of total, animal, and plant protein (based on percentage of kcal/day (*1 kcal=4.18 kJ=0.00418 MJ*) with risk of mortality from all causes, cardiovascular disease (CVD), and cancer in adults aged 19 or older. Dietary intake of protein was modelled with restricted cubic splines in a multivariate random effects dose-response model. Black line indicates the linear model; solid purple line indicates the spline model; dashed lines represent 95% confidence intervals. ES=effect size

Eight 9 10 16 17 18 46 51 64 of 21 publications on the association between total protein intake and all cause mortality were included in the dose-response analysis (fig 5). No significant non-linear association was found (P=0.40 for non-linearity). Furthermore, linear dose-response meta-analysis showed no significant association between total protein intake and all cause mortality by an additional 3% of energy from protein a day (pooled effect size 0.99, 0.97 to 1.00, P=0.10; supplementary fig 1). Combining data from five 10 15 16 18 of 11 papers in the dose-response analysis of animal protein intake and all cause mortality, no significant non-linear association was seen (P=0.54 for non-linearity; fig 5). Moreover, the linear association between an increase of 3% of energy from animal proteins a day and all cause mortality was not significant (pooled effect size 0.99, 0.96 to 1.02, P=0.61; supplementary fig 1). In the dose-response analysis of plant protein intake and all cause mortality, based on six articles^9^ ^10^ ^15^ ^16^ ^18^ ^57^ of 13 publications, a significant non-linear association was found (P=0.05 for non-linearity; fig 5). Based on linear dose-response analysis, an additional 3% of energy from plant proteins a day was associated with a 5% lower risk of death from all causes (pooled effect size 0.95, 95 0.93 to 0.98, P<0.001; supplementary fig 1).

Non-linear dose-response analysis of seven of 10 papers^9^ ^10^ ^16^ ^17^ ^18^ ^51^ ^64^ showed no significant association between intake of total protein and cardiovascular disease mortality (P=0.07; fig 5). Findings from a linear dose response meta-analysis showed no significant association between total protein intake and cardiovascular disease mortality (pooled effect size 0.98, 0.97 to 1.00, P=0.08; supplementary fig 2). No significant non-linear association was found between animal protein intake and cardiovascular disease mortality based on five publications^9^ ^10^ ^15^ ^16^ ^18^ (P=0.37 for non-linearity; fig 5). As with non-linear dose-response meta-analysis, linear dose-response analysis showed no significant association between animal protein intake and cardiovascular disease mortality based on an additional 3% of energy from animal proteins a day (pooled effect size 0.98, 0.94 to 1.02, P=0.32; supplementary fig 2). An inverse association between plant protein intake and cardiovascular disease mortality was found in the non-linear dose-response analysis based on six articles^9^ ^10^ ^15^ ^16^ ^18^ ^57^ (P<0.001 for non-linearity; fig 5). Linear dose-response analysis showed no significant association between an additional 3% of energy from plant protein intake and cardiovascular disease mortality (pooled effect size 0.96, 95 0.89 to 1.04, P=0.30; supplementary fig 2).

Of 13 papers on the association between intake of total protein and cancer mortality, five^9^ ^16^ ^17^ ^18^ ^46^ were included in the non-linear dose-response analysis. No significant association was found between intake of total protein and cancer mortality (P=0.84; fig 5). This was also the case for animal protein (P=0.93) and plant protein intakes (P=0.52) based on four papers^9^ ^15^ ^16^ ^18^ (fig 5). Linear dose-response analysis showed that an additional 3% of energy from total protein intake (pooled effect size 0.98, 0.94 to 1.03, P=0.39), animal protein intake (0.99, 0.96 to 1.02, P=0.50), and plant protein intake (0.94, 0.85 to 1.03, P=0.19) was not associated with cancer mortality (supplementary fig 3).

### Subgroup and sensitivity analyses, and publication bias

To test the robustness of the findings and investigate possible sources of heterogeneity between studies, subgroup analyses were conducted. These analyses were performed based on predefined criteria, including study location, duration of follow-up, sex, dietary assessment tools, health status of study participants, high versus low or middle income countries, single or repeated measurements of protein intake, effect size type, and statistical controlling for confounders (BMI, total energy intake, and macronutrients (fat and carbohydrate)). Supplementary table 3 presents findings for the different subgroups. 

A significant inverse association was seen between total protein intake and all cause mortality in women, in studies that used a food frequency questionnaire for assessment of total protein intake, among those studies that did not control for total energy intake and macronutrients intake, those with a follow up duration of less than 15 years, and those that were performed on people with comorbidities. For cardiovascular disease mortality, a significant inverse association with total protein intake was seen in studies that did not control for total energy intake and macronutrients intake, and among those with a follow-up of less than 15 years. 

For animal protein intake, a significant inverse association was seen with all cause mortality in studies with a follow up duration of less than 15 years. In addition, inverse associations between animal protein intake and mortality from cardiovascular disease were observed in studies that did not control for macronutrients intake. 

Plant protein intake was inversely associated with all cause mortality in both men and women, in studies that were performed in US and non-US countries, in studies with a follow-up of more than 15 years and less than 15 years, in studies that applied a food frequency questionnaire for dietary assessment, among studies that controlled their analysis for energy and macronutrients intakes and BMI, in studies that were done on individuals without comorbidities, in studies performed in high income countries, and in studies that reported a hazard ratio for their analysis. The same findings were also seen between plant protein intake and cardiovascular disease mortality, but this association was not significant in men and women in studies that were performed in the US and those with a follow-up of more than 15 years.

Findings from the sensitivity analysis using a fixed effects model showed that exclusion of the studies by Song et al,^15^ Kurihara et al,^57^ Budhathoki et al,^18^ and Sun et al^11^ resulted in a change in the significant inverse association between plant protein intake and cardiovascular disease mortality to a marginally significant inverse association. Sensitivity analysis for the other associations examined showed that exclusion of any single study from the analysis did not appreciably alter the pooled effect sizes. No missing studies were imputed in regions of the contour enhanced funnel plots. No publication bias was found based on Begg’s rank correlation test. For the association between total protein intake and mortality from all causes and from cardiovascular diseases, and between plant protein intake and mortality from cardiovascular diseases, Egger’s linear regression test indicated possible publication bias. Application of the trim and fill method, however, did not result in a change in the average effect size, further suggesting that the results were not affected by publication bias.

## Discussion

In this systematic review and meta-analysis, we found a significant inverse association between intake of total protein and all cause mortality; no clear significant association was seen between total or animal protein intake and cardiovascular disease and cancer mortality. Intake of plant protein was associated with a lower risk of all cause and cardiovascular disease mortality. The inverse associations between plant protein intake and mortality from all causes and cardiovascular disease remained significant in studies that controlled for energy, BMI, and macronutrients intake, and in studies with follow-up of less than 15 years, and those that applied a food frequency questionnaire for dietary assessment.

### Comparison with other studies

We systematically and quantitatively summarised earlier investigations on the association between intake of total, animal, and plant proteins and mortality. A recent systematic review and meta-analysis showed that consumption of soy protein was significantly associated with a decreased risk of mortality from breast cancer, but it was not associated with mortality from all causes and cardiovascular disease.^19^ In addition, high intake of legumes, grains, and nuts as major sources of plant proteins was associated with a lower risk of all cause and cardiovascular disease mortality.^66^ ^67^ Long term observational studies indicated that high consumption of total and animal proteins was associated with an increased risk of cancer and diabetes.^17^ ^68^ ^69^ Substitution of non-meat proteins for meat proteins has been favourably associated with fasting insulin levels and reduced insulin resistance.^70^ Consumption of low carbohydrate, high protein, and fat diets was not associated with increased risk of coronary heart disease in women. When vegetable sources of fat and protein were chosen, however, these diets were associated with a lower risk of coronary heart disease.^71^ Overall, all available studies support the beneficial effects of plant proteins on human health.

In this meta-analysis, no significant association was seen between animal protein intake and mortality. Unlike our findings, one meta-analysis found that each reduction of three servings of processed meat in a week was associated with a small reduction in the risk of overall cancer mortality over a lifetime.^72^ In addition, fish consumption was associated with a lower risk of all cause mortality among high consumers than among those with the lowest intake.^73^ Thus lack of a significant association between animal protein intake and mortality in our meta-analysis could be due to combining protein from different animal sources, including poultry, eggs, and dairy foods. Also, the discrepant associations of animal meat and animal protein intake. Here, we compared our findings with a previous meta-analysis^72^ on animal meat. In that meta-analysis the exposure variable was meat as a food group, whereas our exposure variable was protein as a nutrient. Animal meat contains fat, sodium, iron, and B vitamins in addition to protein so that those nutrients could affect the risk of mortality differently, whereas animal protein is protein only from animal sources. Therefore, findings for animal meat and animal protein could be different. with mortality explained by the fat content of meat. Some studies investigating the association of animal protein intake and mortality have controlled their analysis for fat intake.^12^ ^15^ ^16^ ^18^ ^58^ ^62^ ^64^ In addition, different methods used in the processing and cooking of meats might provide further explanation for the discrepancy.

In the interpretation of our findings, it must be considered that humans do not consume single macronutrients, such as proteins. Dietary intake of other nutrients and biologically active factors in foods containing protein could also account for the association between protein intake and mortality. In addition, when the contribution of a single nutrient is assessed as a disease risk, the interaction between nutrients in the gut should be taken into account. Some studies included in this meta-analysis had controlled for the confounding effects of other macronutrients (fat or carbohydrates).^7^ ^12^ ^14^ ^15^ ^16^ ^17^ ^18^ ^46^ ^57^ ^58^ ^62^ ^64^ When we confined the analysis to studies that had made these adjustments, the inverse association of plant protein with all cause and cardiovascular disease mortality changed little, whereas the inverse association between intake of total protein and all cause mortality became non-significant. Therefore, dietary fat intake is not likely to account for the protective association between plant protein intake and mortality. Consumption of animal and plant proteins could be a marker of broader dietary intake patterns—or even of social class, an important independent predictor of many health outcomes. Our findings must be interpreted in this context, and future investigations should consider whether intake of animal and plant proteins is a marker of overall dietary patterns or of social class.

### Mechanisms

In this study, intake of plant protein was inversely associated with mortality from all causes and cardiovascular disease. The same finding was also seen for intake of total protein and all cause mortality. Given that plant protein is part of total protein, the observed inverse association for intake of total protein seems to be related to its plant protein component. The mechanisms through which plant proteins could affect human health are not well known. Whereas consumption of animal protein was associated with increased concentrations of insulin-like growth factor 1, dietary intake of plant proteins was not associated with raised levels.^74^ ^75^ Increased levels of insulin-like growth factor 1 have been linked to an increased risk of age related diseases, such as cancers.^76^ ^77^ In addition, dietary plant proteins were associated with favourable changes in blood pressure, waist circumference, body weight, and body composition, which might help to lower the risk of several chronic diseases, including cardiovascular disease and type 2 diabetes.^78^ Intake of animal protein, independent of body weight, was associated with hypercholesterolaemia, whereas consumption of plant proteins was associated with low levels of plasma cholesterol.^79^ ^80^ ^81^

Bacterial fermentation of plant proteins in the gut could help to lower the production of potentially toxic and carcinogenic metabolites, such as ammonia, amines, phenols, tryptophan metabolites, and sulfides.^82^ Bioactive peptides derived from plant proteins could also have beneficial health promoting properties. These proteins and peptides have antioxidative, anti-inflammatory, antihypertensive, and antimicrobial activities.^83^ ^84^ ^85^ Anorectic peptides have been shown to exert their antiobesogenic activity through decreasing food intake.^86^ Earlier studies have indicated that bioactive peptides could reduce blood cholesterol levels.^87^ Furthermore, the different associations between animal or plant proteins and mortality risk could be due to the differences in amino acid composition. Plant proteins contain lower amounts of lysine and histidine amino acids than animal proteins; high intake of these amino acids has been shown to increase secretion of lipoproteins containing apo B.^88^ Therefore, intake of plant proteins could be associated with protection against cardiovascular diseases through this mechanism. In addition to amino acids, plant proteins are rich in non-essential amino acids such as arginine and pyruvate precursors, which in turn can lead to upregulation of glucagon and downregulation of insulin secretion.^89^ The action of glucagon on hepatocytes is mediated through increased cyclic adenosine monophosphate concentrations, which downregulate the synthesis of required enzymes for de novo lipogenesis and upregulate the low density lipoprotein receptors and production of insulin-like growth factor 1 antagonist.^89^

### Strengths and weaknesses of this study

This meta-analysis has several strengths. Firstly, the large number of participants and deaths included allowed us to quantitatively assess the association of protein intake and risk of mortality, thus making it more powerful than any single study. Secondly, a dose-response analysis was conducted to evaluate the linear and non-linear associations. Thirdly, because all the studies included were prospective, the influence of recall and selection bias is negligible. In addition, we considered subtypes of total protein intake, including animal and plant proteins. These data provide a comprehensive insight into the association between intake of dietary protein and risk of mortality based on the current evidence. 

This study has some limitations, most of which are common to observational studies and meta-analyses. Residual or unmeasured confounding factors could have affected the magnitude of the association between protein intake and mortality. Although most studies had controlled for potential confounders, some did not take into account dietary consumption of other nutrients and others did not consider total energy intake and BMI as covariates. Lack of controlling for other nutrients, such as amount and type of dietary fat, which is present in most food sources of protein, could affect the independent association of protein intake with mortality. In addition, some studies in this review did not report sufficient information to be included in the dose-response meta-analysis.****Also, different methods for dietary assessment, including food frequency questionnaires, dietary recall, and records, were used in the included cohorts, and the units of protein intake varied in different studies. Measurement errors in dietary assessment are inevitable and would have tended to underestimate the associations with protein intake. In addition, our conclusions about animal protein intake could have less generalisability to low or middle income economics, in which diets are carbohydrate-rich and consumption of animal sources is low.

### Conclusions, policy implications, and future research

We found that high intake of total proteins was associated with a lower risk of mortality from all causes. Intake of plant protein was also associated with a lower risk of mortality from all causes and cardiovascular diseases, which is consistent with its beneficial effects on cardiometabolic risk factors, including blood lipid and lipoprotein profiles, blood pressure, and glycaemic regulation. These findings have important public health implications as intake of plant protein can be increased relatively easily by replacing animal protein and could have a large effect on longevity. Also, an additional 3% of energy from plant proteins a day was associated with a 5% lower risk of death from all causes. Our findings therefore strongly support the existing dietary recommendations to increase consumption of plant proteins in the general population. Extrapolation of these findings to the worldwide population should be done cautiously because most studies included in the meta-analysis are from Western nations and few studies have been reported from other countries. Therefore, further studies are required. Additional studies should also focus on the mechanisms through which dietary protein affect mortality.

**What is already known on this topic**

Consumption of high protein diets has been suggested to control body weight and improve cardiometabolic abnormalities
Regular consumption of red meat and high intake of animal proteins have been linked to several health problems
Data on the association between different types of proteins and mortality are conflicting

**What this study adds**

High intake of total protein is associated with a lower risk of mortality from all causes
Intake of plant protein is associated with a lower risk of mortality from all causes and from cardiovascular diseases, and an additional 3% of energy from plant proteins a day is associated with a 5% lower risk of death from all causes
These findings support current dietary recommendations to increase consumption of plant proteins in the general population

## Web extra

Extra material supplied by authors

Web appendix: Supplementary material

## Notes

Contributors: OS and SN contributed to the literature search, data extraction, and data analysis. AE contributed to study conception, manuscript drafting, and data analysis. WCW contributed to study conception, manuscript drafting, data analysis, and approving the final manuscript. All authors acknowledge full responsibility for the analyses and interpretation of the report. AE is the guarantor. The corresponding author attests that all listed authors meet authorship criteria and that no others meeting the criteria have been omitted.

Ethical approval: Not required.

Data sharing: No additional data available.

The corresponding author affirms that the manuscript is an honest, accurate, and transparent account of the study being reported. No important aspects of the study have been omitted and any discrepancies from the study as planned have been disclosed.

Dissemination to participants and related patient and public communities: No patients were involved in setting the research question or the outcome measures, nor were they involved in the design and implementation of the study. We plan to disseminate these findings to participants in our annual newsletter and to the general public in a press release.
