# Supplementary material for: Dietary intake of total, animal, and plant proteins and risk of all cause, cardiovascular, and cancer mortality: systematic review and dose-response meta-analysis of prospective cohort studies
Source: BMJ. 2020 Jul 22;370:m2412. doi: 10.1136/bmj.m2412 (PMC7374797; doi:10.1136/bmj.m2412)
Supplement: Supplementary file 1 — Web appendix: Supplementary material [file nags054297.ww.pdf]

Online Supporting Material

Supplemental Table 1: Medical subject headings (MeSH) and non-MeSH keywords used to search relevant publications

**Concept 1**	"dietary protein"[tiab] OR "protein intake"[tiab] OR "plant protein"[tiab] OR "animal protein"[tiab] OR "protein consumption"[tiab] OR "Plant Proteins, Dietary"[Mesh] OR "Dietary Proteins"[Mesh] OR "Fish Proteins, Dietary"[Mesh] OR "Grain Proteins"[Mesh]
**Concept 2**	"Mortality"[tiab] OR "Death"[tiab] OR "fatal"[tiab] OR "Survival"[tiab] OR "Mortality" [Subheading] OR "Neoplasms"[tiab] OR "Cancer Survivors"[tiab] OR “cardiovascular diseases”[tiab] OR “coronary disease”[tiab] OR "Myocardial Ischemia"[tiab] OR “coronary artery disease”[tiab] OR “myocardial infarction”[tiab] OR stroke[tiab] OR mortality[Mesh] OR death[Mesh] OR "Neoplasms"[Mesh] OR "Cancer Survivors"[Mesh] OR “cardiovascular diseases”[Mesh] OR “coronary disease”[Mesh] OR "Myocardial Ischemia"[Mesh] OR “coronary artery disease”[Mesh] OR “myocardial infarction”[Mesh] OR stroke[Mesh]

The combination of keywords as mentioned above was used to search online databases.

("concept 1" AND "concept 2")

Online Supporting Material

Supplemental Table 2: Results of risk of bias assessment based on the ROBINS-E tool

Author	Bias due to confounding	Bias in selection of participants into the study	Bias in classification of exposures	Bias due to departures from intended exposures	Bias due to missing data	Bias in measurement of outcomes	Bias in selection of the reported result	Overall judgment
Virtanen et al. 2019	Low	Low	Low	Low	Low	Low	Low	Low
Alonso et al. 2015	Low	Low	Low	Low	Low	Low	Low	Low
Song et al. 2016	Low	Low	Low	Low	Low	Low	Low	Low
Zaslavsky et al. 2017	Low	Low	Low	Low	Low	Low	Low	Low
Dehghan et al. 2017	Low	Low	Low	Low	Low	Low	Low	Low
Courand et al. 2016	Moderate	Low	Low	Low	Low	Low	Low	Moderate
Argos et al. 2013	Low	Low	Low	Low	Low	Low	Low	Low
Kelemen et al. 2005	Low	Low	Low	Low	Low	Low	Low	Low
Payette et al. 1999	Critical	Moderate	Moderate	Moderate	Low	Low	Low	Critical
Papanikolaou et al. 2019	NI	Low	Low	NI	NI	Low	Low	Low
Sun et al. 2019	NI	Low	Low	NI	NI	Low	Low	Low
Levine et al. 2014	Low	Low	Low	Low	Low	Low	Low	Low
Budhathoki et al. 2019	Low	Low	Low	Low	Low	Low	Low	Low
Halbesma et al. 2009	Moderate	Low	Low	Low	Low	Low	Low	Moderate
Holmes et al. 1999	Low	Low	Low	Low	Low	Low	Low	Low
Mendonca et al. 2019	Moderate	Moderate	Moderate	NI	Low	Low	Low	Moderate
Chan et al. 2019	Low	low	Low	Low	Low	Low	Moderate	Moderate
Dwyer et al. 1994	Serious	Low	Low	Moderate	Low	Low	Moderate	Serious
Song et al. 2019	Low	Low	Low	Low	Low	Low	Low	Low
Bates et al. 2010	Serious	Low	Low	Moderate	Low	Low	Low	Serious
Kuijpers et al. 2015	Low	Low	Low	Low	Moderate	Low	Low	Moderate
Peis et al. 2010	Low	Low	Low	Low	Low	Low	Low	Low
Sauvaget et al. 2004	Moderate	Low	Low	Low	Low	Low	Low	Moderate
Nagata et al. 2015	Low	Low	Low	Low	Low	Low	Moderate	Moderate
Esrey et al. 1996	Low	Low	Low	Low	Low	Low	Moderate	Moderate
Smit et al. 2007	Low	Low	Low	Low	Moderate	Low	Low	Moderate
Holmes et al. 2017	Low	Low	Low	Low	Moderate	Low	Low	Moderate

Borugian et al. 2004	Moderate	Low	Low	Moderate	Low	Low	Low	Moderate
Rohan et al. 1993	Low	Moderate	Low	Low	Low	Low	Low	Moderate
Kurihara et al. 2018	Moderate	Low	Low	Low	Low	Low	Low	Moderate
Palli et al. 2000	Moderate	Moderate	Low	Moderate	Low	Low	Low	Moderate
Tharrey et al.2018	Low	Low	Low	Low	Low	Low	Low	Low

Abbreviations: NI: no information, ROBINS-E: risk of bias in non-randomized studies of exposures

The risk of bias in non-randomized studies of exposures (ROBINS-E) tool comprises 7 domains through which bias might be introduced. The questions of these domains include: (1) bias due to confounding, (2) bias in selection of participants into study, (3) bias in the classification of exposures, (4) bias due to departure from intended exposures, (5) bias due to missing data, (6) bias in the measurement of outcomes, and (7) bias in the selection of reported results. Studies were categorized as low risk, moderate risk, serious risk, and critical risk of bias under each domain.

Supplemental Table 3: Stratified analysis on associations of total, animal, and plant protein intakes with risk of mortality from all-cause, CVD, and cancer in adults aged ≥18 years

	Total protein				Animal protein				Plant protein			
	n	ES (95% CI)[1]	I^2 (%)[2]	P[3]	n	ES (95% CI)[1]	I^2 (%)[2]	P[3]	N	ES (95% CI)[1]	I^2 (%)[2]	P[3]
**All-cause mortality**												
Location												
US	6	0.99 (0.98 to 1.01)	60.1	0.02	4	1.01 (1.00 to 1.03)	0	0.53	5	0.97 (0.95 to 0.99)	76.7	0.002
Non US	14	0.96 (0.90 to 1.02)	58.2	0.002	7	0.98 (0.91 to 1.05)	57.9	0.01	8	0.88 (0.82 to 0.95)	0	0.45
Sex												
Male and female	12	0.99 (0.97 to 1.01)	52.2	0.01	6	1.01 (1.00 to 1.02)	62.6	0.02	7	0.97 (0.96 to 0.99)	69.9	0.003
Male	4	1.06 (0.93 to 1.20)	58.6	0.06	4	1.03 (0.89 to 1.18)	39.5	0.17	4	1.02 (0.86 to 1.20)	0	0.83
Female	7	0.78 (0.68 to 0.89)	41.5	0.11	3	0.91 (0.74 to 1.13)	24.9	0.26	4	0.91 (0.87 to 0.95)	24.7	0.26
Follow-up duration												
>15 years	6	1.00 (0.98 to 1.02)	0	0.64	6	1.01 (1.00 to 1.03)	0	0.50	7	0.97 (0.95 to 0.98)	67.7	0.003
<15 years	15	0.86 (0.80 to 0.92)	54.3	0.005	5	0.83 (0.72 to 0.97)	52.4	0.06	6	0.89 (0.80 to 0.99)	32.4	0.18
Dietary assessment tools:												
FFQ	13	0.96 (0.93 to 0.99)	49.8	0.01	7	1.01 (0.97 to 1.05)	46.1	0.06	8	0.90 (0.87 to 0.93)	0	0.46
Food recall and record	6	1.00 (0.98 to 1.02)	57.7	0.03	4	1.01 (1.00 to 1.03)	57.5	0.07	5	0.99 (0.97 to 1.01)	9	0.35
Adjustment for energy												
Yes	16	0.97 (0.94 to 1.00)	50.9	0.007	9	1.01 (0.97 to 1.06)	39	0.08	9	0.90 (0.85 to 0.94)	0	0.46
No	4	0.81 (0.69 to 0.95)	67.6	0.02	1	0.45 (0.23 to 0.89)	-	-	2	0.87 (0.76 to 1.00)	0	0.45
Unclear	1	1.00 (0.99 to 1.02)	-	-	1	1.01 (1.00 to 1.03)	-	-	2	0.98 (0.96 to 1.00)	89.4	0.002
Adjustment for BMI												
Yes	15	0.97 (0.94 to 1.00)	58.7	0.001	9	1.01 (0.97 to 1.05)	53.7	0.01	10	0.90 (0.86 to 0.94)	0	0.51
No	5	0.91 (0.82 to 1.00)	51.7	0.04	1	1.09 (0.83 to 1.44)	-	-	1	0.82 (0.62 to 1.09)	-	-
Unclear	1	1.00 (0.99 to 1.02)	-	-	1	1.01 (1.00 to 1.03)	-	-	2	0.98 (0.96 to 1.00)	89.4	0.002
Health status												
Healthy	15	0.99 (0.97 to 1.01)	51.7	0.01	9	1.01 (1.00 to 1.02)	33.3	0.13	11	0.97 (0.95 to 0.98)	60.5	0.001
Unhealthy	6	0.81 (0.70 to 0.95)	62.6	0.02	2	0.91 (0.72 to 1.16)	84	0.01	2	0.91 (0.71 to 1.17)	57.9	0.12
Adjustment for fat and CHO												
Yes	7	0.99 (0.96 to 1.03)	0	0.49	6	1.02 (0.98 to 1.06)	48.8	0.06	7	0.89 (0.85 to 0.94)	0	0.78
No	13	0.83 (0.77 to 0.90)	47.3	0.02	4	0.88 (0.75 to 1.03)	42.3	0.14	4	0.90 (0.76 to 1.06)	38.7	0.16
Unclear	1	1.00 (0.98 to 1.02)	-	-	1	1.01 (1.00 to 1.03)	-	-	2	0.98 (0.96 to 1.00)	89.4	0.002
High-income country												
Yes	19	0.99 (0.97 to 1.01)	59.5	<0.001	11	1.01 (1.00 to 1.02)	45.2	0.03	13	0.97 (0.95 to 0.98)	57.5	0.003
No	1	1.07 (0.85 to 1.35)	-	-	0	-	-	-	0	-	-	-

Assessment of protein intake												
Single measurement	20	0.99 (0.97 to 1.01)	60.1	0.001	10	1.01 (0.99 to 1.02)	48.2	0.03	12	0.97 (0.95 to 0.99)	51.5	0.01
Repeated measurements	1	0.98 (0.94 to 1.02)	-	-	1	1.03 (0.98 to 1.08)	-	-	1	0.89 (0.83 to 0.95)	-	-
Effect size type												
HR	17	0.99 (0.97 to 1.01)	58.5	0.001	9	1.01 (1.00 to 1.02)	50.8	0.02	11	0.97 (0.95 to 0.98)	63	0.001
RR	3	0.90 (0.75 to 1.09)	59.5	0.06	1	0.82 (0.59 to 1.13)	-	-	1	0.95 (0.82 to 1.10)	-	-
OR	1	1.32 (0.81 to 2.16)	-	-	1	1.01 (0.52 to 1.96)	-	-	1	1.19 (0.66 to 2.14)	-	-
**CVD mortality**												
Location												
US	3	1.01 (0.99 to 1.02)	0	0.61	3	1.01 (1.00 to 1.03)	19.7	0.28	4	0.98 (0.96 to 1.01)	82.5	0.001
Non US	6	0.92 (0.81 to 1.04)	24.1	0.23	5	0.98 (0.84 to 1.15)	43.1	0.10	6	0.83 (0.75 to 0.93)	0	0.73
Sex												
Male and female	7	1.01 (0.99 to 1.02)	48.1	0.07	5	1.01 (1.00 to 1.03)	63.2	0.02	6	0.98 (0.96 to 1.01)	73.4	0.002
Male	2	0.95 (0.71 to 1.26)	0	0.39	2	0.98 (0.68 to 1.41)	13.5	0.28	2	0.90 (0.62 to 1.32)	0	0.93
Female	3	0.95 (0.73 to 1.25)	0	0.76	3	1.10 (0.78 to 1.55)	0	0.64	4	0.85 (0.78 to 0.93)	0	0.63
Follow-up time												
>15 years	5	1.01 (0.99 to 1.02)	0	0.95	5	1.01(1.00 to 1.03)	0	0.57	6	0.98 (0.96 to 1.00)	76.1	<0.001
<15 years	5	0.82 (0.69 to 0.98)	24.9	0.24	3	0.78 (0.54 to 1.13)	59.5	0.06	4	0.87 (0.77 to 0.99)	0	0.67
Dietary assessment tools												
FFQ	8	0.97 (0.91 to 1.02)	0	0.79	6	1.07 (0.99 to 1.16)	0	0.56	7	0.86 (0.81 to 0.91)	0	0.68
Food recall and record	2	1.01 (0.99 to 1.02)	82.3	0.01	2	1.01 (0.99 to 1.02)	81.8	0.01	3	1.00 (0.97 to 1.02)	55.6	0.10
Adjustment for energy												
Yes	7	0.97 (0.92 to 1.03)	0	0.90	6	1.07 (0.99 to 1.16)	0	0.56	6	0.85 (0.78 to 0.92)	0	0.60
No	2	0.68 (0.50 to 0.93)	52.8	0.14	1	0.45 (0.23 to 0.89)	-	-	2	0.87 (0.76 to 1.00)	0	0.45
Unclear	1	1.01 (1.00 to 1.03)	-	-	1	1.01 (1.00 to 1.03)	-	-	2	0.99 (0.97 to 1.01)	87.7	0.004
Adjustment for BMI												
Yes	9	0.96 (0.91 to 1.02)	4.6	0.39	7	1.06 (0.97 to 1.15)	33.4	0.15	8	0.85 (0.79 to 0.91)	0	0.72
No	0	-	-	-	-	-	-	-	-	-	-	-
Unclear	1	1.01 (1.00 to 1.03)	-	-	1	1.01 (1.00 to 1.03)	-	-	2	0.99 (0.97 to 1.01)	87.7	0.004
Health status												
Healthy	8	1.01 (0.99 to 1.02)	0	0.43	7	1.01 (1.00 to 1.03)	22.6	0.24	9	0.97 (0.95 to 0.99)	66	0.001
Unhealthy	2	0.82 (0.60 to 1.14)	62.9	0.10	1	2.54 (0.87 to 7.42)	-	-	1	1.73 (0.56 to 5.33)	-	-
Unclear	0	-	-	-	-	-	-	-	-	-	-	-
Adjustment for fat and CHO												
Yes	5	0.98 (0.93 to 1.04)	0	0.87	5	1.08 (0.99 to 1.17)	0	0.50	6	0.85 (0.79 to 0.91)	0	0.51
No	4	0.80 (0.67 to 0.96)	2.4	0.39	2	0.67 (045 to 0.99)	5.9	0.34	2	0.91 (0.61 to 1.35)	0	0.66
Unclear	1	1.01 (0.99 to 1.02)	-	-	1	1.01 (1.00 to 1.03)	-	-	2	0.99 (0.97 to 1.01)	87.7	0.004
High-income country												
Yes	9	1.01 (0.99 to 1.02)	18.8	0.26	8	1.01 (0.99 to 1.03)	37.2	0.12	10	0.97 (0.95 to 0.99)	63.7	0.001

No	0	-	-	-	0	-	-	-	0	-	-	-
Assessment of protein intake												
Single measurement	9	1.01 (0.99 to 1.02)	19.9	0.25	7	1.01 (0.99 to 1.02)	25.9	0.21	9	0.98 (0.96 to 1.00)	61.1	0.004
Repeated measurements	1	0.98 (0.92 to 1.05)	-	-	1	1.09 (0.99 to 1.20)	-	-	1	0.88 (0.80 to 0.97)	-	-
Effect size type												
HR	9	1.01 (0.99 to 1.02)	22.7	0.22	7	1.01 (1.00 to 1.03)	38.6	0.11	9	0.97 (0.95 to 0.99)	62.8	0.003
RR	1	0.84 (0.39 to 1.08)	-	-	1	0.88 (0.42 to 1.85)	-	-	1	0.70 (0.49 to 0.99)	-	-
**Cancer mortality**												
Location												
US	5	1.00 (0.98 to 1.02)	0	0.76	5	1.00 (0.98 to 1.02)	0	0.48	5	1.00 (0.97 to 1.04)	0	0.51
Non US	7	0.96 (085 to 1.08)	61.3	0.01	4	0.97 (0.86 to 1.11)	23.1	0.26	4	0.97 (0.85 to 1.10)	40.3	0.15
Sex												
Male and female	6	1.00 (0.98 to 1.02)	18.8	0.29	5	1.00 (0.98 to 1.03)	0	0.63	5	1.00 (0.96 to 1.03)	16.8	0.30
Male	2	0.92 (0.66 to 1.30)	74.4	0.04	2	0.79 (0.54 to 1.16)	0	0.37	2	1.24 (0.86 to 1.78)	0	0.86
Female	5	0.87 (0.73 to 1.04)	47.9	0.10	3	0.83 (0.69 to 1.00)	0	0.90	3	1.02 (0.87 to 1.19)	50.3	0.13
Follow-up time												
>15 years	5	1.00 (0.98 to 1.02)	0	0.96	5	1.00 (0.98 to 1.02)	0	0.47	5	1.00 (0.97 to 1.04)	0	0.54
<15 years	7	0.95 (0.79 to 1.30)	63.9	0.007	4	0.99 (0.80 to 1.21)	23	0.26	4	0.89 (0.73 to 1.09)	31.1	0.21
Dietary assessment tools												
FFQ	10	0.97 (0.92 to 1.03)	45.5	0.05	7	0.99 (0.92 to 1.05)	18.7	0.28	7	0.97 (0.89 to 1.04)	21.2	0.26
Food recall and record	2	1.00 (0.98 to 1.02)	17.9	0.27	2	1.00 (0.98 to 1.03)	0	0.97	2	1.01 (0.97 to 1.05)	0	0.58
Adjustment for energy												
Yes	11	0.98 (0.93 to 1.03)	44.4	0.04	8	0.99 (0.92 to 1.05)	7.2	0.37	8	0.97 (0.90 to 1.05)	14.5	0.31
No	-	-	-	-	-	-	-	-	-	-	-	-
Unclear	1	1.00 (0.98 to 1.02)	-	-	1	1.00 (0.98 to 1.03)	-	-	1	1.01 (0.97 to 1.05)	-	-
Adjustment for BMI												
Yes	9	0.98 (0.93 to 1.04)	32.5	0.14	7	0.98 (0.92 to 1.05)	13.4	0.32	7	0.98 (0.91 to 1.06)	11.4	0.34
No	2	0.88 (0.68 to 1.14)	82.6	0.01	1	1.09 (0.83 to 1.04)	-	-	1	0.82 (0.62 to 1.09)	-	-
Unclear	1	1.00 (0.98 to 1.02)	-	-	1	1.00 (0.98 to 1.03)	-	-	1	1.01 (0.97 to 1.05)	-	-
Health status												
Healthy	7	1.00 (0.98 to 1.02)	34.8	0.15	6	1.00 (0.98 to 1.02)	0	0.62	6	1.00 (0.97 to 1.04)	18.6	0.28
Unhealthy	5	0.94 (0.80 to 1.10)	55.4	0.06	3	0.96 (0.81 to 1.13)	51.8	0.12	3	0.96 (0.81 to 1.15)	25.9	0.25
Adjustment for fat and CHO												
Yes	5	0.99 (0.94 to 1.05)	35.3	0.18	4	1.01 (0.94 to 1.08)	7	0.35	4	0.97 (0.89 to 1.05)	0	0.54
No	6	0.90 (0.87 to 1.03)	49.3	0.06	4	0.90 (0.77 to 1.05)	0	0.45	4	0.98 (0.84 to 1.14)	44.2	0.12
Unclear	1	1.00 (0.98 to 1.02)	-	-	1	1.00 (0.98 to 1.03)	-	-	1	1.01 (0.97 to 1.05)	-	-
High-income country												
Yes	11	1.00 (0.98 to 1.02)	33.7	0.12	9	1.00 (0.98 to 1.02)	0	0.45	9	1.00 (0.97 to 1.04)	12.2	0.33
No	1	1.79 (0.99 to 3.24)	-	-	-	-	-	-	-	-	-	-

Assessment of protein intake												
Single measurement	11	1.00 (0.98 to 1.02)	45	0.04	8	1.00 (0.97 to 1.02)	5.7	0.38	8	1.01 (0.97 to 1.05)	0	0.44
Repeated measurements	1	0.98 (0.92 to 1.05)	-	-	1	1.02 (0.94 to 1.11)	-	-	1	0.92 (0.82 to 1.03)	-	-
Effect size type												
HR	8	1.00 (0.98 to 1.02)	33.9	0.14	6	1.00 (0.98 to 1.02)	0	0.47	6	1.00 (0.96 to 1.03)	35	0.16
RR	3	0.91 (0.75 to 1.09)	66.4	0.05	2	0.84 (0.69 to 1.02)	0	0.72	2	1.07 (0.91 to 1.25)	0	0.77
OR	1	1.32 (0.81 to 2.16)	-	-	1	1.01 (0.52 to 1.96)	-	-	1	1.19 (0.66 to 2.14)	-	-

Abbreviation: ES: effect size- HR: hazard ratio- OR: odds ratio- RR: risk ratio- CI: confidence interval- FFQ: food frequency questionnaire- BMI: body mass index- US: united states- CVD: cardiovascular diseases, CHO: carbohydrate

[1]Obtained from the fixed-effects model; these effect sizes are for the highest versus lowest comparison

[2]Inconsistency- percentage of variation across studies due to heterogeneity

[3]Obtained from the Q-test.

Online Supporting Material
Supplemental Figure 1

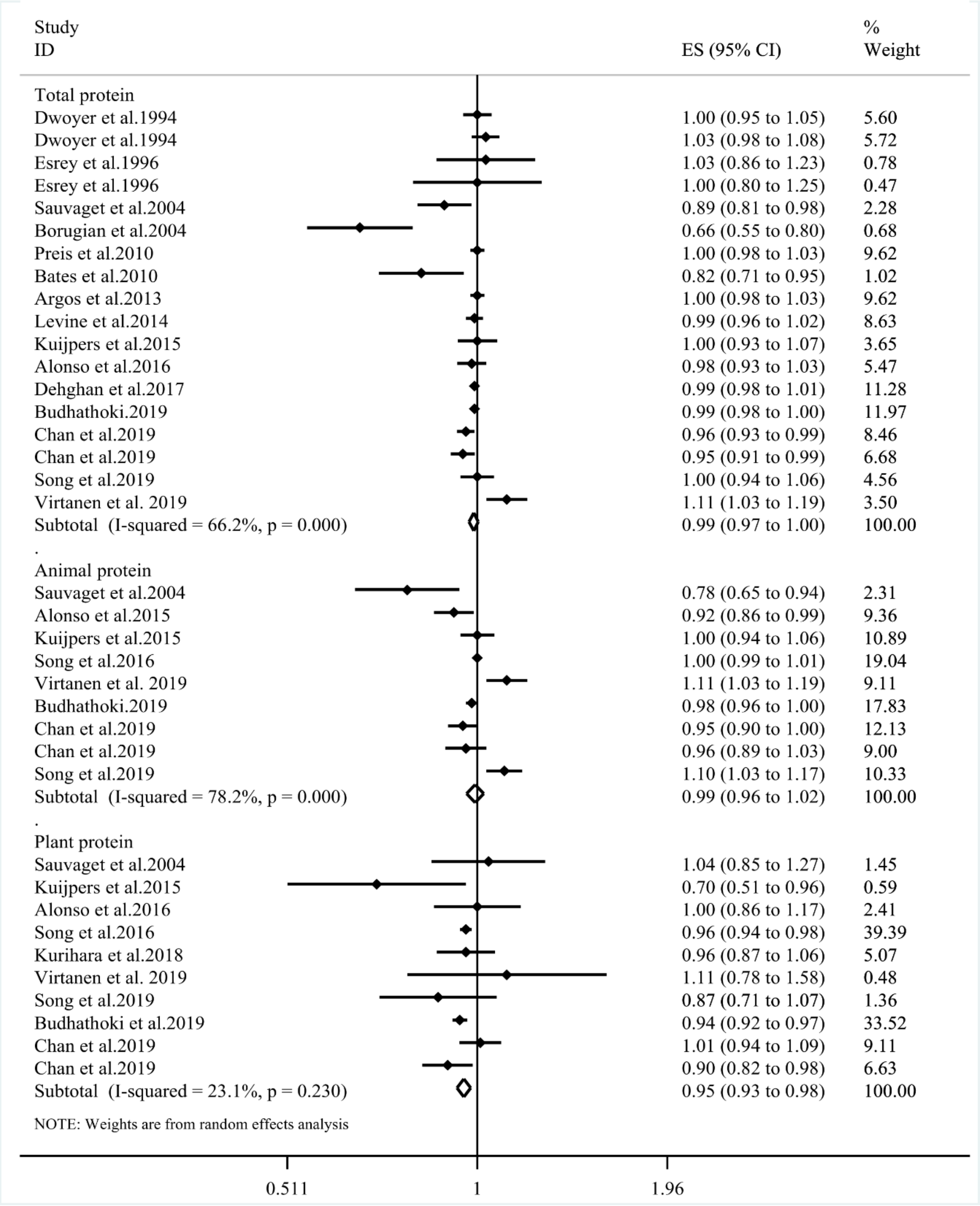

Forest plot for the risk of all-cause mortality based on 3% increase in energy from protein intake in adults aged >18 years. Horizontal lines represent 95% CIs. Diamonds represent the pooled estimates from the random-effects analysis. ES: effect size, CI: confidence interval

Online Supporting Material
Supplemental Figure 2

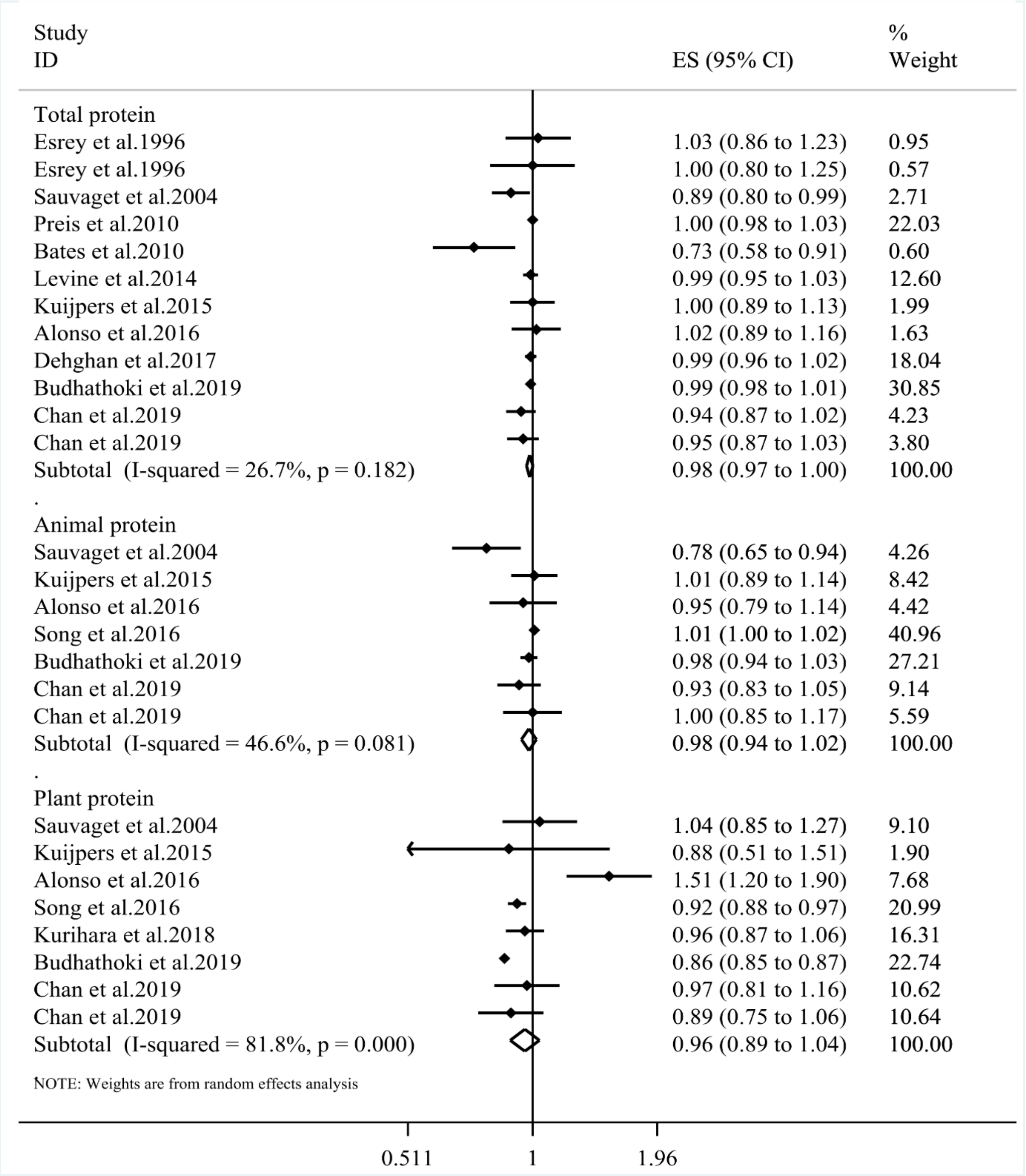

Forest plot for the risk of CVD mortality based on 3% increase in energy from protein intake in adults aged >18 years. Horizontal lines represent 95% CIs. Diamonds represent the pooled estimates from the random-effects analysis. ES: effect size, CI: confidence interval

Online Supporting Material
Supplemental Figure 3

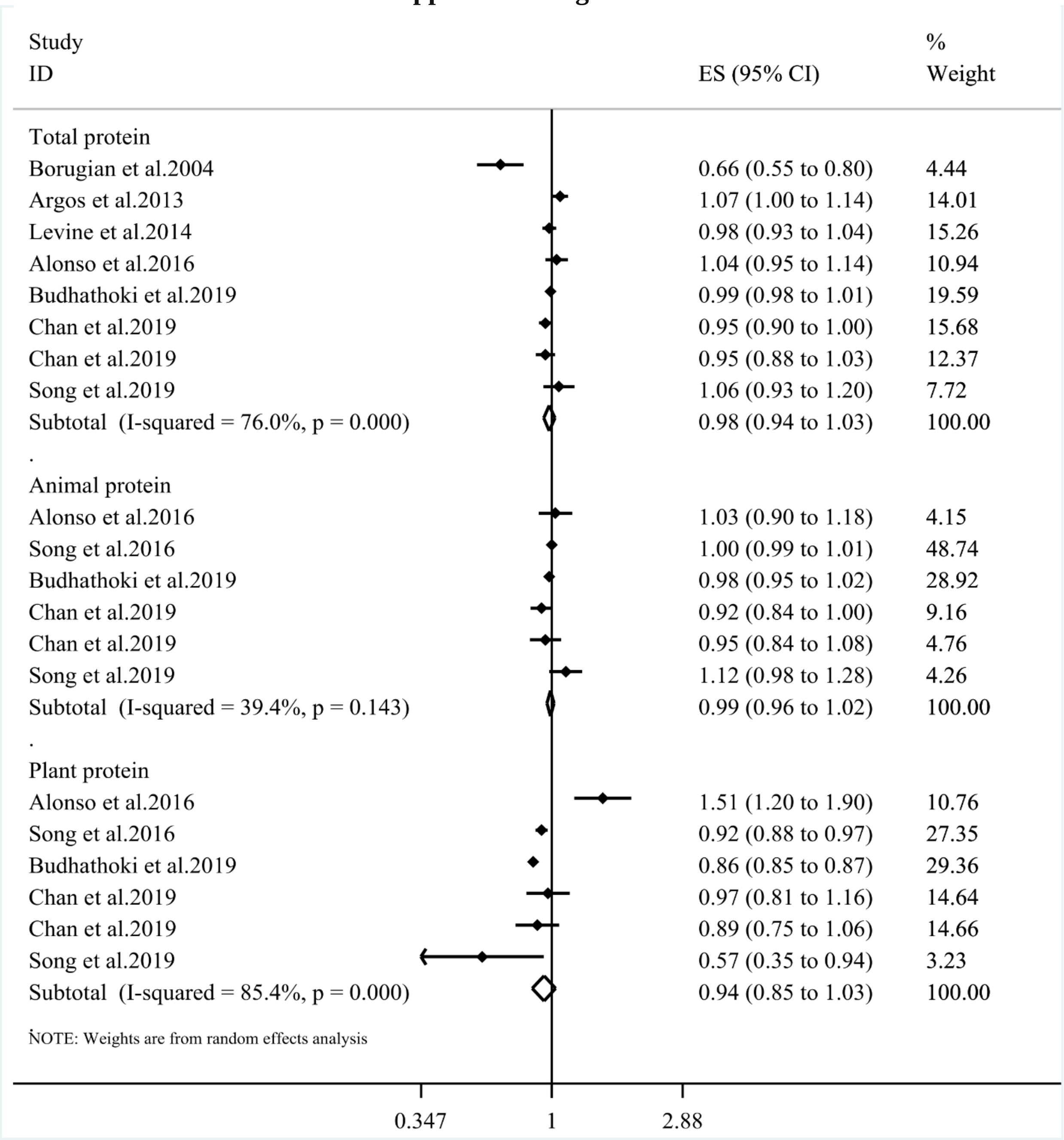

Forest plot for the risk of cancer mortality based on 3% increase in energy from protein intake in adults aged >18 years. Horizontal lines represent 95% CIs. Diamonds represent the pooled estimates from the random-effects analysis. ES: effect size, CI: confidence interval
